# Offspring production of haploid spermatid-like cells derived from mouse female germline stem cells with chromatin condensation

**DOI:** 10.1186/s13578-021-00697-z

**Published:** 2022-01-04

**Authors:** Xiaopeng Hu, Hu Wang, Geng. G. Tian, Changliang Hou, Bo Xu, Xinyan Zhao, Yongqiang Zhao, Qian Fang, Xinyue Li, Lin He, Xuejin Chen, Shangang Li, Ji Wu

**Affiliations:** 1 Bio-X Institutes, Shanghai Jiao Tong University, No. 800. Dongchuan Road, Minhang District, Shanghai, 200240 China; 2 State Key Laboratory of Primate Biomedicine Research, Institute of Primate Translational Medicine, Kunming University of Science and Technology, Kunming, 650500 China; 3 Key Laboratory of Fertility Preservation and Maintenance of Ministry of Education, Ningxia Medical University, Yinchuan, China; 4 Department of Laboratory Animal Science, Shanghai Jiao Tong University School of Medicine, 280 South Chongqing Road, Shanghai, 200025 China; 5 Shanghai Key Laboratory of Reproductive Medicine, Shanghai, 200025 China; 6 Yunnan Key Laboratory of Primate Biomedicine Research, Institute of Primate Translational Medicine, Kunming University of Science and Technology, Kunming, 650500 China

**Keywords:** Female germline stem cells, Transdifferentiation, In vitro spermatogenesis, Spermatid-like cells, High-throughput chromosome conformation capture sequencing, Bivalent

## Abstract

**Background:**

During male meiosis, the Y chromosome can form perfect pairing with the X chromosome. However, it is unclear whether mammalian Female germline stem cells (FGSCs) without a Y chromosome can transdifferentiate into functional haploid spermatid-like cells (SLCs).

**Results:**

We found that spermatogenesis was restarted by transplanting FGSCs into *Kit*^*w/wv*^ mutant testes. Complete meiosis and formation of SLCs was induced in vitro by testicular cells of *Kit*^*w/wv*^ mutant mice, cytokines and retinoic acid. Healthy offspring were produced by sperm and SLCs derived from the in vivo and in vitro transdifferentiation of FGSCs, respectively. Furthermore, high-throughput chromosome conformation capture sequencing(Hi-C-seq) and “bivalent” (H3K4me3-H3K27me3) micro chromatin immunoprecipitation sequencing (μChIP-seq) experiments showed that stimulated by retinoic acid gene 8 (STRA8)/protamine 1 (PRM1)-positive transdifferentiated germ cells (tGCs) and male germ cells (mGCs) display similar chromatin dynamics and chromatin condensation during in vitro spermatogenesis.

**Conclusion:**

This study demonstrates that sperm can be produced from FGSCs without a Y chromosome. This suggests a strategy for dairy cattle breeding to produce only female offspring with a high-quality genetic background.

**Supplementary Information:**

The online version contains supplementary material available at 10.1186/s13578-021-00697-z.

## Introduction

Spermatogenesis depends on the stem cell niche and microenvironment created by testicular somatic cells and cytokines. These cells and cytokines promote the self-renewal of spermatogonial stem cells (SSCs), male meiosis, and spermiogenesis through cell signal transduction. For example, SSCs injected into *Kit* mutant sterile mice can develop into spermatozoa [1, 2]; SSCs co-cultured in vitro with testicular somatic cells, melatonin, and cytokines can develop into functional spermatozoa [3].

Many studies have shown that the microenvironment and epigenetics are closely related and that they interact with each other. Feil et al*.* suggested that the microenvironment can influence gene expression and regulate cell fate through epigenetic changes [4]. Changes in gene expression induced by the microenvironment are often accompanied by epigenetic changes, such as DNA methylation and histone modification, which enable cells to respond rapidly to environmental changes [5]. For example, the metabolic cycle is closely linked to epigenetic changes in the tumor microenvironment. Enzymes that regulate epigenetics can promote DNA transcription and post-translational modifications, thereby affecting the expression of metabolism-related genes [6]. Changes in epigenetic status can also affect the secretion of various hormones and cytokines, which alter the microenvironment, eventually affecting cell fate such as proliferation, survival, and differentiation [4].

In the process of spermatogenesis, epigenetic characteristics change substantially [7]. Mammalian germ cells undergo several key transformations during gametogenesis involving tightly regulated chromatin remodeling, which resets the balanced chromatin structure [8]. In particular, topologically associating domains (TADs) undergo dissolution and reconstruction during spermatogenesis [8, 9]. Interestingly, in humans and mice, mature germ cell nuclear bodies (usually with H3K4me3 and H3K27me3 modifications) are found at promoters of hundreds of genes that are important for spermatogenesis [7]. H3K4me3 (related to gene activation) and H3K27me3 (related to gene silencing) are “bivalent” and most bivalent promoters are hypomethylated in embryonic stem cells and sperm. Recently, bivalent nucleosomes have been localized in mouse spermatocytes and spermatids [10, 11]. However, during in vitro spermatogenesis, the variation of three-dimensional chromosome structures and bivalent histone modifications has not yet been tested.

During male meiosis, the Y chromosome can form perfect pairing with the X chromosome. Evolution has shortened the Y chromosome, so that it is now only one-third of the length of the X chromosome, and contains fewer than 50 genes [12]. If this trend continues, the Y chromosome may disappear altogether. Is it possible to produce new male individuals without a Y chromosome? In fact, the Y chromosome has been lost by a few mammalian species over the course of evolution [13, 14]. The X chromosome of most clinical XX males contains the sex-determining region of the Y chromosome (*SRY*), which may have resulted from chromosome translocation [15]. *SRY* on the Y chromosome upregulates the expression of *SRY*-box transcription factor 9 (*SOX9*) to determine sex [16]. Transfer of *Eif2s3y* and *Sry*, two Y chromosome genes, into XX female mouse embryos was reported to produce males [17]. Knockout of a small segment of *enh13*, a distal enhancer of *Sox9*, causes sex reversal [18]. *Sox9* is expressed in testicular Sertoli cells but is barely expressed in other testis cells. Sertoli cells in vivo maintain the ability to transdifferentiate into their opposite-sex counterparts [19]. A small number of clinical XX males do not have the *SRY* gene on the X chromosome but carry mutations in genes that affect the transition of granulosa cells into Sertoli cells, such as Wilms tumor 1 [15, 20]. An interesting question is whether female germline stem cells (FGSCs) with an XX karyotype can undergo male meiosis in the testicular microenvironment to produce functional sperm.

Here, we found that FGSCs isolated from mouse ovaries can develop into functional sperm through epigenetic changes regulated by the testicular microenvironment. These results indicate that, in mice, FGSCs without a Y chromosome can complete male meiosis and develop into functional sperm under the influence of the testicular microenvironment created by testicular somatic cells, retinoic acid (RA), and cytokines. This helps address the key question of whether sperm can be produced from FGSCs that do not possess a Y chromosome, and indicates a strategy for dairy cattle breeding to produce only female offspring with a high-quality genetic background.

## Results

### In vivo spermatogenesis from FGSCs

To observe whether FGSCs in a testis microenvironment can transdifferentiate into early-stage male germ cells, we injected GFP-labeled FGSCs into testis seminiferous tubules and cultured these tubules in vitro [21]. After culture for 2–15 days, the seminiferous tubules were dissected and observed by fluorescence microscopy. Immunofluorescence analysis showed that the injected GFP-positive cells were located in tubules and that the relative number of GFP-positive cells per tubule increased with continuing culture (Additional file 1: Figure S3A and S4A). We then characterized promyelocytic leukemia zinc finger (PLZF) (an SSC marker [22]) and GFP by dual immunofluorescence analysis, and we observed GFP-PLZF double-positive cells distributed throughout the tubules. The relative number of GFP-PLZF double-positive cells per tubule also increased as the testis culture continued (Additional file 1: Figure S3B and S4B). These results indicated that FGSCs in seminiferous tubules can transdifferentiate into SSCs or early-stage male germ cells.

**Fig. 1:**
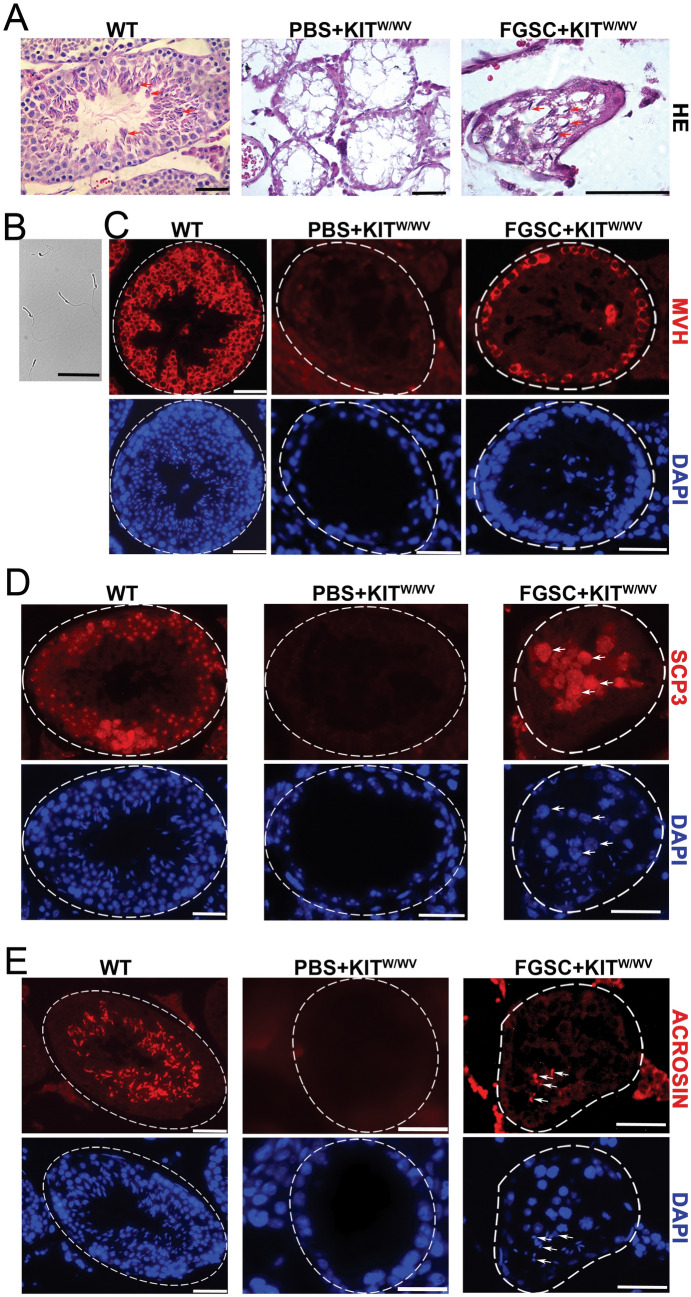
Investigation of spermatogenesis after transplantation of FGSCs into *Kit*^*w/wv*^ mouse seminiferous tubules. **A** The recipients were analyzed by H&E staining at 6–8 weeks after transplantation. **B** Sperm were isolated from the epididymal tail of recipient mice at 6–8 weeks after transplantation. **C** Spermatogenesis was analyzed by MVH immunofluorescence 6–8 weeks after transplantation. **D** Meiosis was confirmed by SCP3 immunofluorescence 6–8 weeks after transplantation. **E** Sperm were produced and confirmed by ACR immunofluorescence 6–8 weeks after transplantation. Scale bar, 50 µm. WT: wild type

To investigate whether FGSCs can transdifferentiate into functional sperm or restart spermatogenesis in a recipient testis in vivo, the GFP-labeled FGSCs were transplanted into the seminiferous tubules of recipient *Kit*^*w/wv*^ mutant mice (6–8 weeks old) which are sterile because of germ cell deficiency. 6 to 8 weeks after FGSC transplantation, sperm were found in some recipient seminiferous tubules (sperm indicated by arrows; Fig. 1A). To observe sperm morphology, we removed sperm from the epididymal tail (Fig. 1B). Moreover, the occurrence of meiosis in recipient seminiferous tubules was confirmed by the immunofluorescent detection of mouse vasa homologue (MVH) -positive germ cells (Fig. 1C), synaptonemal complex protein 3 (SCP3, a component of the synaptonemal complex [23])-positive spermatocytes (Fig. 1D), and acrosin (ACR)-positive sperm (Fig. 1E). In *Kit* mutant testes, however, spermatogenesis was arrested and no differentiating germ cells were found, in contrast to the findings in wild-type testes (Fig. 1A and C–E). These results collectively indicate that transplanted FGSCs can transdifferentiate into sperm in recipient *Kit*^*w/wv*^ testis by restarting spermatogenesis in recipient seminiferous tubules.

### Offspring production of sperm derived from transplanted FGSCs

**Fig. 2:**
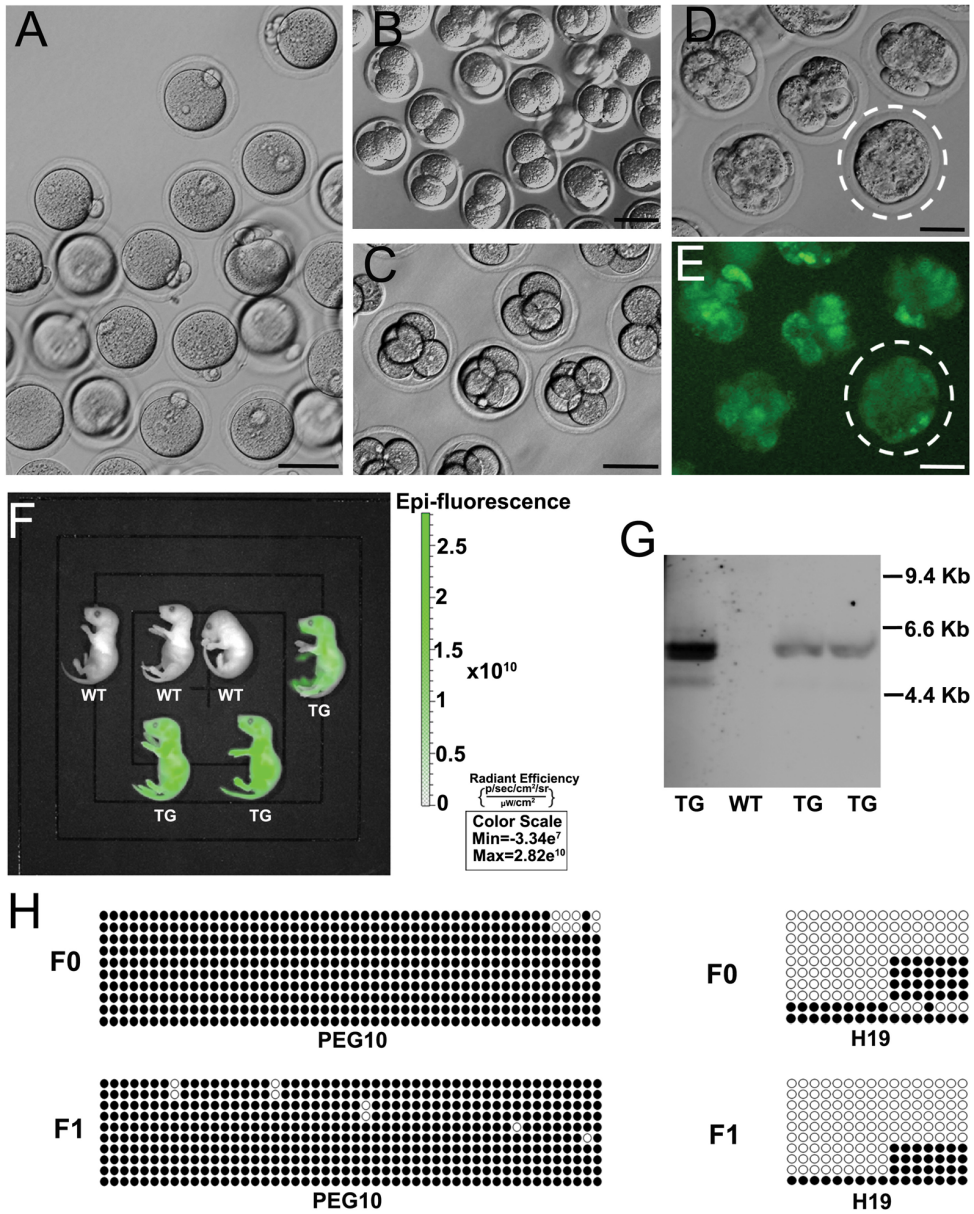
Full functionality of sperm in vivo-derived from FGSCs was assessed by ICSI. Zygotes (**A**), two-cell embryos (**B**), four-cell embryos (**C**), and GFP signals in blastocysts (**D**, **E**) were observed in in vitro culture (KSOM_AA_ medium) after ICSI with sperm isolated from the epididymal tail of recipient mice. **F** Strong green fluorescence signals in the offspring detected by live imaging under a Lumazone imaging system (MagBiosystems). TG: transgenic mice; WT: wild type. **G** GFP sequence in the offspring detected by Southern blot analysis. TG: transgenic mice; WT: wild type. **H** The methylation status of the DMRs of *Peg10* and *H19* genes in the mother (F_0_) and offspring (F_1_), as shown by bisulfite sequencing analysis. Scale bar, 50 µm

To confirm the full functionality of sperm derived from the in vivo transdifferentiation of FGSCs, Intracytoplasmic sperm injection (ICSI) was performed. ICSI-administered sperm were able to activate oocytes, and zygotes developed to two-cell and four-cell embryos and blastocysts in culture in KSOM_AA_ medium (Fig. 2A–E). After the transfer of FGSC-derived two-cell embryos to the oviduct, we obtained three full-term pups that were transgenic for actin-GFP. Strong green fluorescence signals were detected in the offspring by live imaging under a Lumazone imaging system (MagBiosystems) (Fig. 2F). Moreover, Southern blot analysis confirmed the GFP transgenic nature of the offspring (Fig. 2G). There were no marked fluctuations in the methylation status [differentially methylated regions (DMRs) of paternally expressed gene 10 (*Peg10*) and *H19*] between parent and offspring, as revealed by bisulfite sequencing analysis (Fig. 2H).

### Induction of spermatogenesis from FGSCs to haploid spermatid-like cells in vitro

**Fig. 3:**
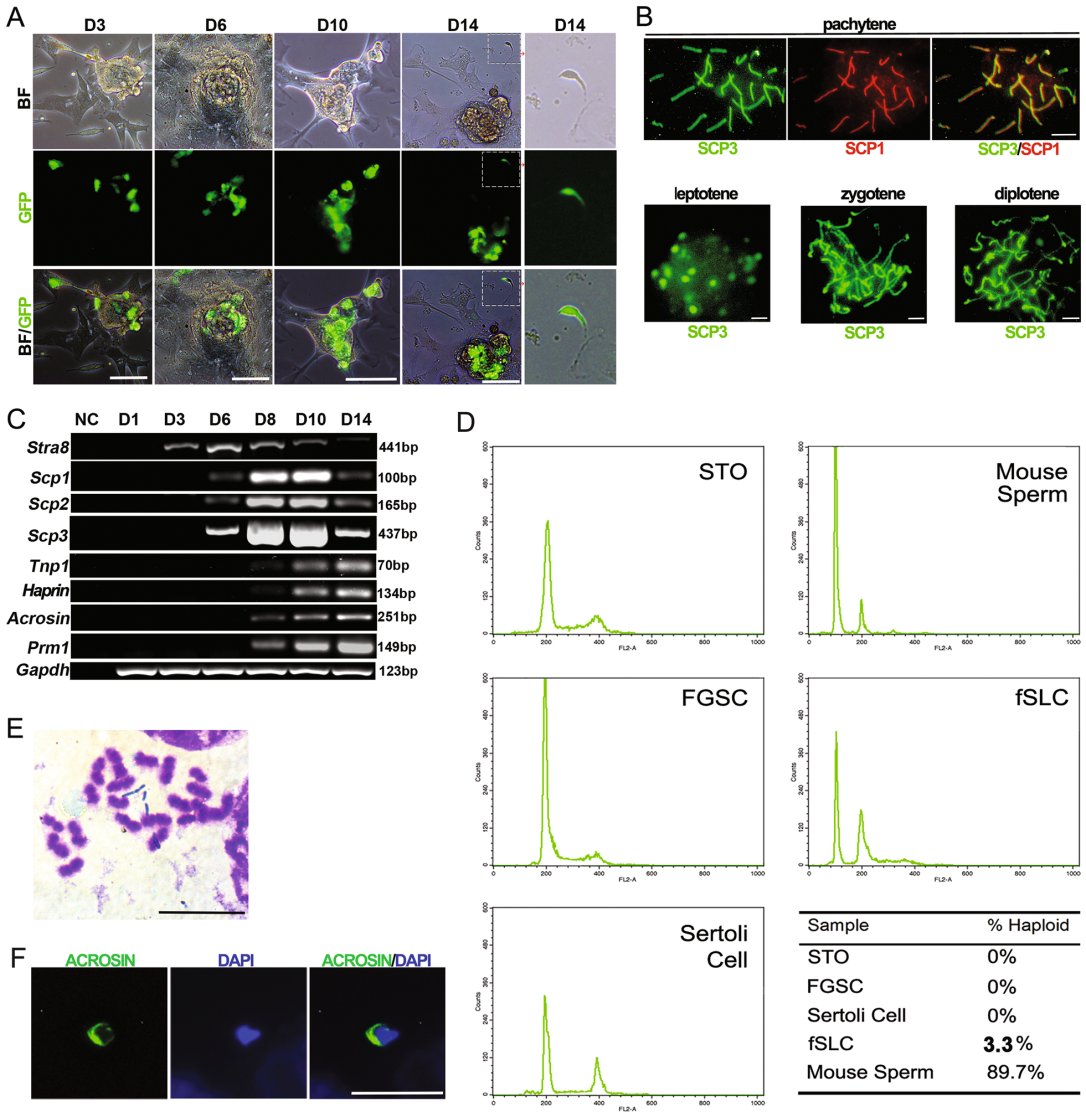
Induction of meiosis and formation of haploid SLCs during in vitro transdifferentiation of FGSCs. **A** FGSCs isolated from actin-GFP mouse ovaries were induced by *Kit*^*w/wv*^ testicular cells, RA, and cytokines. This process was observed at different time points. **B** Chromosomal synapsis and different stages of meiosis (leptotene, zygotene, pachytene, diplotene) were assessed by immunofluorescence of SCP1 and SCP3. **C** Transcripts of meiotic factors and haploid spermatid markers were detected by RT-PCR at different time points. **D** The DNA content of fSLCs was determined by flow cytometry analysis. The rate of FGSCs underwent complete meiosis was the number of fSLCs at Day 14 divided by the number of FGSCs at Day 0 and then divided by 2. **E** Haploid cells were confirmed by karyotype analysis. **F** fSLCs were confirmed by ACR immunofluorescence. Scale bar, 50 µm

On the basis of the above results, we investigated whether FGSCs have the potential to transdifferentiate into SLCs in vitro. FGSCs isolated from actin-GFP mice were cultured for eight passages, mixed with *Kit*^*w/wv*^ testicular cells, and co-cultured for 6 days in MEMα supplemented with KSR, activin A, BMPs-2/4/7, and RA (Fig. 3A). On day 7 of co-culture, we withdrew the culture supernatant fluid and exposed the FGSC-derived cultures to combinations of MEMα, KSR, FSH, BPE, and T (Fig. 3A). To observe the progression of meiosis induced in vitro, we assessed chromosomal synapsis by immunofluorescence of synaptonemal complex protein 1 (SCP1) and SCP3 in the transdifferentiated cells. Characteristic appearances of different meiotic stages (leptotene, zygotene, pachytene, and diplotene) were observed, as shown in Fig. 3B. These results indicated that synapsis had occurred in the transdifferentiated cells. Consistent with this, we found that transcripts of meiotic factors were upregulated in a programmed manner, similar to one cycle of meiotic division in vivo. Transcripts of *Stra8*, a marker of meiotic initiation, were first detectable on day 3, upregulated to the highest levels on day 6, and then decreased by day 14 (Fig. 3C). Transcripts of *Scp3* were first detectable on day 6, upregulated to the highest levels on day 10, and then decreased sharply on day 14, consistent with progression to the diplotene stage. Transcripts of haploid spermatid markers, such as *Prm1*, *Tnp1*, *Haprin*, and *Acr*, were first detectable on day 8 and were upregulated to the highest levels on day 14 (Fig. 3C). To analyze whether haploid cells were produced, the DNA content of SLCs derived from FGSCs (fSLCs) was determined by Fluorescence Activating Cell Sorter (FACS) analysis. As shown in Fig. 3D, a haploid population of fSLCs was produced corresponding to haploid peaks of positive (mouse sperm) and negative (STO cells, FGSCs, and Sertoli cells) controls. Induced by testicular cells of *Kit* mutant mice and in basic medium plus morphogens, approximately 3.3% of FGSCs underwent complete meiosis and were transdifferentiated into haploid cells (Fig. 3D). Furthermore, karyotype analysis confirmed the existence of haploid cells from in vitro transdifferentiation (Fig. 3E) and the fSLCs exhibited ACR localization (Fig. 3F). These results indicated that FGSCs can be induced by testicular cells, RA, and cytokines to transdifferentiate into SLCs in vitro.

### Offspring production of spermatid-like cells in vitro-derived from FGSCs

**Fig. 4:**
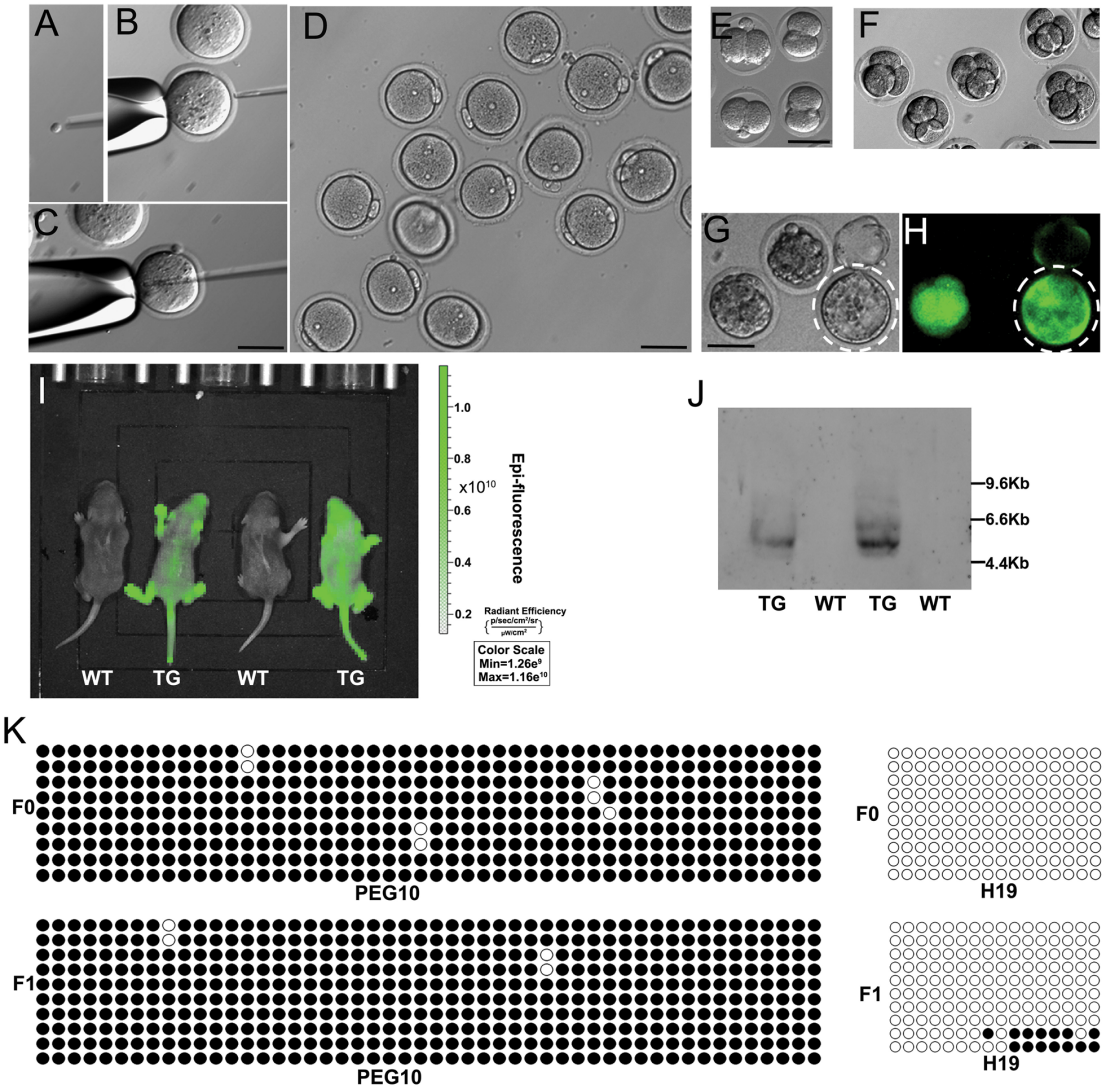
Production of healthy fertile offspring with in vitro-derived fSLCs. **A–C** The process of ICSI with fSLCs. Zygotes (**D**), two-cell embryos (**E**), four-cell embryos (**F**), and GFP signals in blastocysts (**G**, **H**) were observed in in vitro culture (KSOM_AA_ medium) after ICSI with fSLCs derived from in vitro transdifferentiation. **I** Fluorescence was detected by live imaging under a Lumazone imaging system (MagBiosystems) and strong green fluorescence signals in offspring were shown. TG: transgenic mice; WT: wild type. **J** GFP sequence in the offspring detected by Southern blot analysis. TG: transgenic mice; WT: wild type. **K** The methylation status of the DMRs of *Peg10* and *H19* genes in the mother (F_0_) and offspring (F_1_), as shown by bisulfite sequencing analysis. Scale bar, 50 µm

We next performed ICSI with FACS-sorted in vitro-derived fSLCs (Fig. 4A–C). By ICSI, fSLCs were able to activate oocytes, and zygotes developed to two-cell embryo, four-cell embryo, and blastocyst stages in culture with KSOM_AA_ medium (Fig. 4D–H). Of 50 oocytes injected with FGSC-derived SLCs, 17 developed to the two-cell stage after activation and five GFP-transgenic blastocysts were obtained after culture in KSOM_AA_ medium. Of 65 oocytes injected with FGSC-derived SLCs, 23 developed to the two-cell stage after activation and two GFP-transgenic pups were obtained after two-cell embryo transfer. Strong green fluorescence signals in the offspring were detected by live imaging under a Lumazone imaging system (MagBiosystems) (Fig. 4I). Moreover, Southern blot analysis showed a GFP sequence in the offspring, but not in the control mice (Fig. 4J). There were no marked fluctuations in the methylation status (DMRs of *Peg10* and *H19*) between parent and offspring, as revealed by bisulfite sequencing analysis (Fig. 4K).

### Comparison of chromatin dynamics between FGSCs and SSCs during in vitro spermatogenesis

To compare key stages during the in vitro transdifferentiation of FGSCs with those of SSCs, we used FGSC/SSC lines expressing EGFP or EBFP controlled by the cell-specific *Stra8* [24] or the *Prm1* promoter [25, 26], respectively. Only in MEMα supplemented with KSR, activin A, BMPs-2/4/7, and RA did *Stra8*-EGFP-expressing female germ cells (STRA8-tGCs) become apparent within 3 days of exposure (Additional file 1: Figure S6A), but *Stra8*-EGFP-expressing male germ cells (STRA8-mGCs) became apparent within 1 day (Additional file 1: Figure S6A). In the presence of MEMα containing KSR/FSH/T/BPE, post-meiotic *Prm1*-EBFP-expressing female germ cells (PRM1-tGCs) produced strong reporter expression on day 14 (Additional file 1: Figure S6B), but *Prm1*-EBFP-expressing male germ cells (PRM1-mGCs) became apparent on day 12 (Additional file 1: Figure S6B). The in vitro differentiation process of mouse SSCs is shown in Additional file 1: Figure S5. During this process, we analyzed the expression of germ-cell-specific markers by RT-PCR and immunofluorescence.

To reveal changes of chromosome conformation and to better understand the dynamics of epigenetic regulation during in vitro transdifferentiation, we performed Hi-C-seq and ChIP-seq for the H3K27me3 mark (which is associated with facultatively repressed promoters) and the H3K4me3 mark (which is associated with active promoters) [27] on germ cells at each stage (STRA8-mGCs, PRM1-mGCs, STRA8-tGCs, and PRM1-tGCs). Germ cells at each stage were sorted by flow cytometry, based on being positive for EGFP or EBFP. Original Hi-C-seq or H3K27me3 and H3K4me3 data for SSCs and FGSCs were obtained from previously published articles [28–30].

**Fig. 5:**
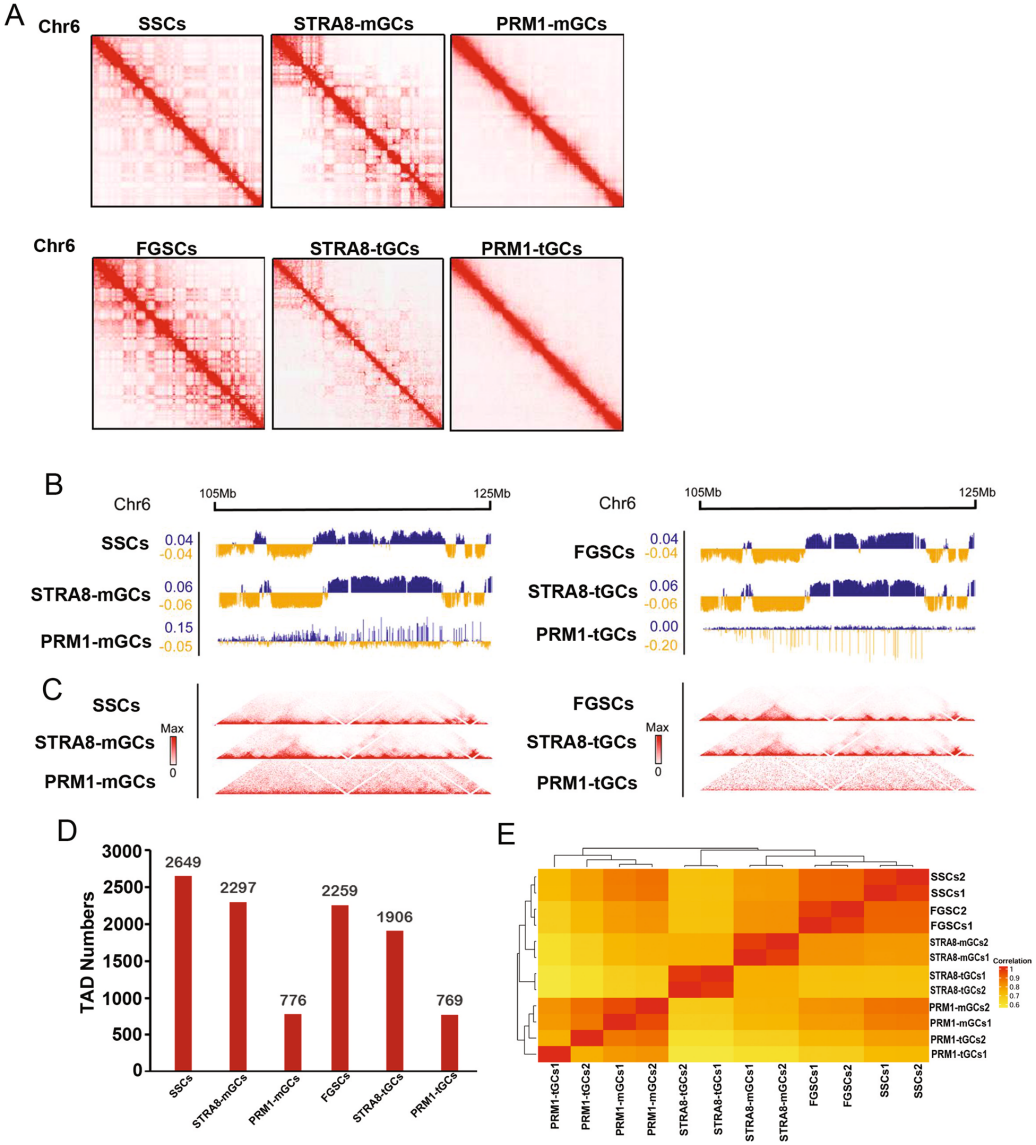
Overall chromosome structure in different cell states. **A** Heatmap showing normalized Hi-C interaction contacts of chromosome 6 in different cell states. **B**, **C** A/B compartment-indicated principal component 1 value and normalized Hi-C interaction contacts displayed at 20 kb resolution. **D** Numbers of TADs in different cell states. **E** The correlation of Hi-C interactions between replicates

For each stage, we sequenced 8.0–9.0 billion Hi-C reads, resulting in 73–386 million pairwise chromatin contacts (Additional file 1: Table S2). The comparison between biological replicates resulted in highly reproducible Hi-C maps (Additional file 1: Figure S7A). As shown in Fig. 5, genome organization changed during in vitro spermatogenesis and transdifferentiation. At the initiation of meiosis (STRA8-SSCs or STRA8-FGSCs), there was little difference in the status of compartments and the numbers of TADs of the two cell lines were similarly decreased, compared with those of undifferentiated SSCs or FGSCs, respectively (Fig. 5B). The pattern of the A/B compartment signal dramatically changed in both PRM1-mGCs and PRM1-tGCs and the signals of both were weaker than in the previous two stages (Fig. 5B). The numbers of TADs in both PRM1-mGCs and PRM1-tGCs were decreased compared with those at the previous two stages (totals of 776 and 769 TADs in PRM1-mGCs and PRM1-tGCs genomes, respectively) (Fig. 5C and D). Additionally, correlation analysis of Hi-C interactions between each replicate also showed that chromatin dynamics was similar between FGSCs and SSCs throughout in vitro spermatogenesis (Fig. 5E).

**Fig. 6:**
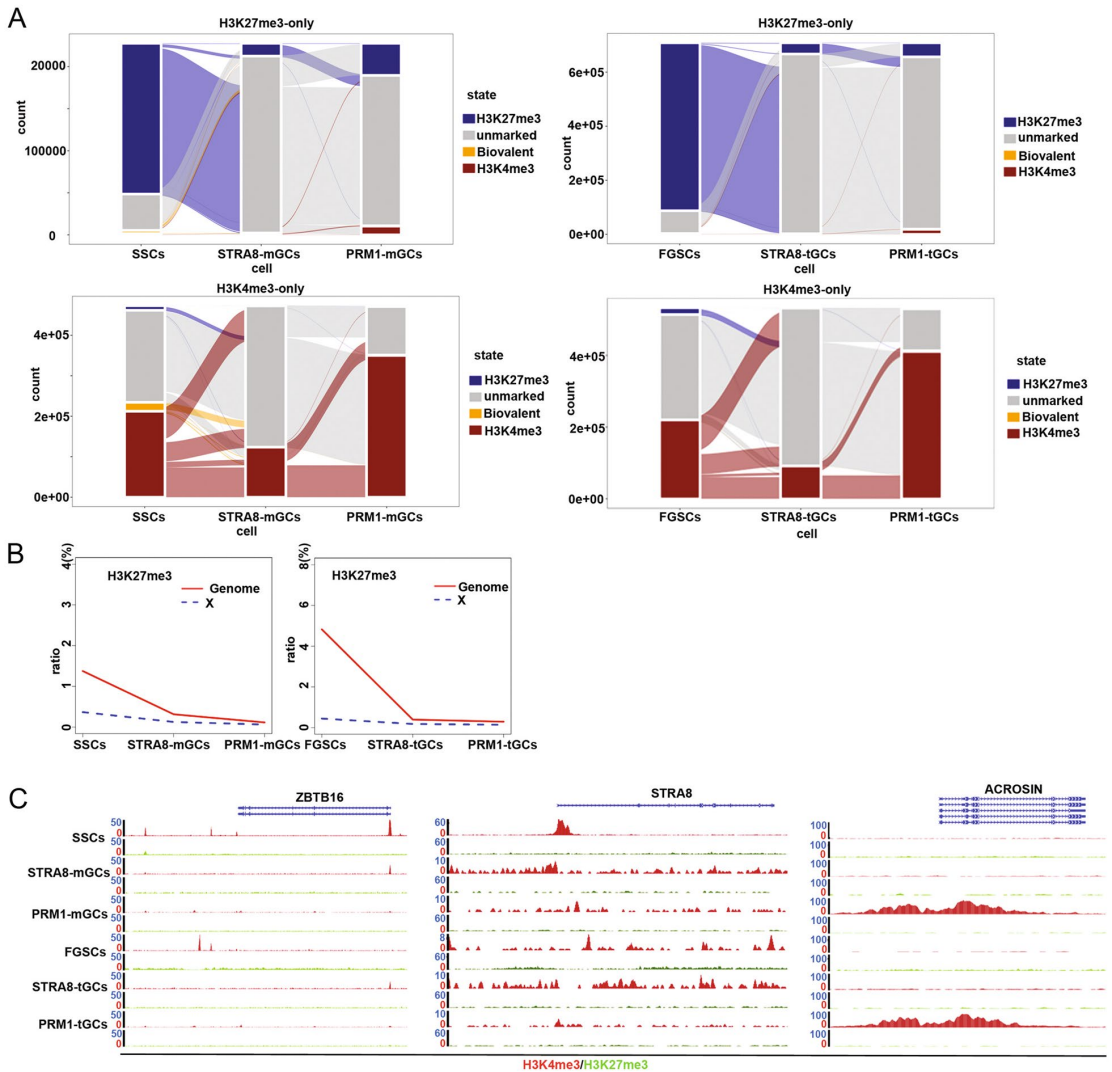
Histone modification of H3K4me3 and H3K27me3 in different cell states. **A** Different cell states had global dynamics of H3K27me3-only and H3K4me3-only regions, which defined ChromHMM categories in 200 bp bin. **B** The H3K27me3 histone modification dynamics were ploted with solid line and chromosome X dynamics were plotted dashed line. **C** A snapshot of the IGV genome browser showing the reads of H3K4me3 and H3K27me3 for *Plzf*, *Stra8*, and *Acr* in different cell states

Epigenetic modifications are highly dynamic and change extensively during in vivo spermatogenesis [7, 11]. However, genome-wide histone modifications during in vitro spermatogenesis remain unclear. To compare genome-wide histone modifications between FGSCs and SSCs during in vitro spermatogenesis, we mapped H3K4me3 and H3K27me3 modifications from undifferentiated SSCs and FGSCs to post-meiosis (SSCs, STRA8-mGCs, PRM1-mGCs, FGSCs, STRA8-tGCs, and PRM1-tGCs). Comparisons between biological replicates resulted in highly reproducible ChIP-seq data (Additional file 1: Figure S7B). To analyze the chromatin state throughout the transdifferentiation of FGSCs into male haploid cells and in the in vitro spermatogenesis of SSCs, we performed ChromHMM analysis. All regions of the genome were classified based on the profiles of two histone modifications: H3K4me3-only and H3K27me3-only regions. In this process, the global number of H3K4me3-only regions in both STRA8-mGCs and STRA8-tGCs dramatically decreased compared with that at the undifferentiated stage (SSC and FGSC, respectively) (Fig. 6A). However, the global number of H3K4me3-only regions in both PRM1-mGCs and PRM1-tGCs increased compared with that at the early meiosis-initiated or undifferentiated stage (Fig. 6A). Similarly, in both the transdifferentiation of FGSCs into male haploid cells and the in vitro spermatogenesis of SSCs, the number of H3K27me3-only regions tended to decrease first and then to increase with development (Fig. 6A). We also found a continuous decrease of H3K27me3 on the X chromosome from undifferentiated SSCs or FGSCs to the post-meiosis stage (PRM1-mGCs or PRM1-tGCs) (Fig. 6B). Interestingly, transcriptional start sites (TSSs) of the zinc finger and BTB domain containing 16 (*Zbtb16*) gene in SSCs but not in FGSCs were marked with H3K4me3, which is consistent with PLZF expression in SSCs but not in FGSCs (Fig. 6C). However, the TSSs of *Zbtb16* in both STRA8-tGCs and STRA8-mGCs were marked with a weaker H3K4me3 signal than in SSCs (Fig. 6C). The TSSs of *Zbtb16* in both PRM1-tGCs and PRM1-mGCs were marked with a weak H3K4me3 signal (Fig. 6C). The promoter of *Stra8* (1.4 kb) in both STRA8-tGCs and STRA8-mGCs had a clear H3K4me3 signal compared with the findings in the other two stages of respective cells (Fig. 6C). The promoter or TSSs of *Acr* in both PRM1-tGCs and PRM1-mGCs had a strong H3K4me3 signal compared with the findings in the other two stages of respective cells (Fig. 6C). These results indicated that H3K4me3 and H3K27me3 modifications robustly separate the three stages of FGSC transdifferentiation into male haploid cells or in vitro SSC spermatogenesis.

To examine genome-wide chromatin states across developmental time points, we treated all six conditions (two cell lines and three developmental stages) as equivalent, and used *k*-means clustering to group all genes according to the similarity of H3K4me3 and H3K27me3 profiles near their TSSs (Additional file 1: Figure S8A). Clusters 1 and 2 were significantly enriched for functional categories corresponding to DNA repair (Additional file 1: Figure S8B); cluster 3 was significantly enriched for functional categories corresponding to meiosis, synapsis, and spermiogenesis (Additional file 1: Figure S8B). These results indicated that FGSCs and SSCs underwent meiosis and spermiogenesis, induced by testicular somatic cells, RA, and cytokines.

Interestingly, when *k*-means clustering analysis and Hi-C-seq data were combined, the number of B compartments in all clusters increased and the number of A compartments in all clusters decreased with development (for both SSCs and FGSCs) (Additional file 1: Figure S9A). The number of genes inside TADs of all clusters increased and the number of genes outside TADs of all clusters decreased with development (for both SSCs and FGSCs) (Additional file 1: Figure S9B). An increase in the number of B compartments causes an increase in the heterochromatin ratio, and an increase in the number of genes in TADs is associated with a decrease in the number of enhancers and a decrease in gene expression. Taken together, these results indicate that, as meiosis progresses in vitro spermatogenesis, chromosomes gradually condense, leading to the silencing of many genes.

### Methylation and expression analysis of imprinted genes in sperm-like cells derived from FGSCs in vitro

To evaluate the epigenetic status of SLCs transdifferentiated from FGSCs in vitro, DMRs of a maternally imprinted region (*Igf2r*) and a paternally imprinted region (*H19*) were examined using bisulfite sequencing analysis. The maternally imprinted *Igf2r* DMR showed complete methylation but there was no methylation of the paternally imprinted *H19* DMR in FGSCs (Additional file 1: Figure S10A). As expected, the major *Igf2r* and *H19* DMR sites in fSLCs were demethylated and methylated, respectively, although a few *Igf2r* DMR and *H19* DMR sites were still methylated and unmethylated, respectively. The methylation status of fSLCs was similar to that of sperm (Additional file 1: Figure S10A), which were unmethylated in the *Igf2r* DMR and methylated in the *H19* DMR. Furthermore, the expression of maternally *(Dlk1*, *Plagl1*, and *Snrpn*) and paternally (*H19*, *Gtl2*, and *Grb10*) imprinted genes was detected. Our analysis revealed that maternally imprinted gene expression increased and paternally imprinted gene expression decreased during the culture period (Additional file 1: Figure S10B–C). These observations indicated that epigenetic transition of imprinted gene methylation and expression was associated with fSLCs transdifferentiated from FGSCs.

## Discussion

FGSCs can self-renew and differentiate into functional oocytes [31–41]. Here, after the injection of FGSCs into the testes of *Kit* mutant mice, fertile sperm were produced in the epididymal tail of recipient mice at six to eight weeks after FGSCs transplantation (Additional file 2: Graphic abstract). Induced by *Kit* mutant testis cells, RA, and cytokines, FGSCs can also undergo complete meiosis to produce SLCs at day 14, and healthy offspring can be produced after ICSI (Additional file 2: Graphic abstract). Furthermore, to obtain a better understanding of how chromatin regulates the process of SSC differentiation and FGSC transdifferentiation in vitro, we investigated changes in chromatin structure (by Hi-C-seq) or histone modification (H3K4me3 and H3K27me3 by ChIP-seq) in the meiosis initiation stage (STRA8^+^) and the post-meiotic stage (PRM1^+^) (Additional file 2: Graphic abstract), which were not characterized in previous studies [7, 8, 11]. Hi-C-seq and ChIP-seq analysis showed that FGSCs and SSCs displayed similar chromatin dynamics and chromatin condensation across developmental time points of in vitro spermatogenesis (Additional file 2: Graphic abstract). Bisulfite sequencing analysis showed that the methylation and expression of imprinted genes transitioned during the transdifferentiation of FGSCs into SLCs.

The results of our study indicate that somatic cells of the testis and cytokines provide a microenvironment to change the epigenetic status and the gene expression of FGSCs, induce meiosis, and eventually force their development into male haploid cells. Indeed, germ cell differentiation is controlled by the gonad somatic environment or sexual identity of the gonad proper. Notably, microenvironment signals, including growth factors (paracrine and circulating) and extracellular matrix, as well as cell-to-cell contacts or interactions with Sertoli cells, may play an important role in FGSC transdifferentiation [42]. After the injection of FGSCs into the testis of *Kit* mutant mice, the injected FGSCs expressed PLZF, a marker of undifferentiated SSCs. We also investigated changes to the chromatin structure (by Hi-C-seq) and histone modification (H3K4me3 and H3K27me3 by ChIP-seq) during SSC differentiation and FGSC transdifferentiation in vitro, both of which had similar epigenetic dynamics. To investigate whether the methylation and expression of imprinted genes changed during the transdifferentiation of FGSCs into SLCs, we examined key imprinted genes, including *Dlk1*, *Plagl1*, *Snrpn*, *H19*, *Gtl2*, and *Grb10*, and showed that the expression of these genes switched following the transdifferentiation of FGSCs into SLCs.

H3K4me3 may play a critical role in spermiogenesis in our in vitro transdifferentiation system. We found that the global number of H3K4me3-only regions in both PRM1-mGCs and PRM1-tGCs increased compared with that in early meiosis-initiated or undifferentiated stages. Moreover, the promoter region or TSSs of *Acr* in both PRM1-tGCs and PRM1-mGCs was strongly marked with an H3K4me3 signal compared with the findings in the other two stages of the respective cells.

We found that most A/B compartments had disappeared from, and most TADs were dissociated in, PRM1-mGCs and PRM1-tGCs. We did not find that A/B compartments reappeared or that TADs underwent apparent reorganization in PRM1-SSCs and PRM1-FGSCs. However, during in vivo spermatogenesis, the A/B compartments and TADs underwent blurry reappearance after being lost in previous stages and a large reorganization of genome structure occurred in the post-meiosis round sperm stage [8, 9]. There are two possible reasons for this. One is that *Prm1*-positive cells were probably widely distributed in the zygotene, pachytene, diplotene, and post-meiosis stages [43], which results in a reduced haploid ratio of *Prm1*-positive cells. Second, we used approximately 10,000 cells to perform Hi-C-seq; this is different from the previous study, in which a large number of cells were used, resulting in the Hi-C-seq data of our study not reaching very high resolution. Therefore, we did not see significant reconstructions of A/B compartments and TADs.

These results indicate that FGSCs underwent spermatogenesis rather than oogenesis. When induced by *Kit* mutant testis cells, RA, and cytokines, FGSCs can also undergo complete meiosis and characteristic stages of meiosis (leptotene, zygotene, pachytene, diplotene) were observed by SCP3 and SCP1 immunofluorescence. Both spermatogenesis and oogenesis involve meiosis, but they are very different epigenetically. In our study, both STRA8-mGCs and STRA8-tGCs, as well as PRM1-tGCs and PRM1-mGCs, had highly similar A/B compartments, TADs, and histone modification (H3K4me3 and H3K27me3). Haploid cells, which were derived from FGSCs induced by *Kit* mutant testis cells, RA, and cytokines, were identified as haploid spermatids by the prominent expression of *Prm1*, *Tnp1*, *Haprin*, and *Acr* on day 14. Moreover, the methylation status of fSLCs was comparable to that of sperm. The major *Igf2r* and *H19* DMRs in fSLCs were demethylated and methylated, respectively.

In the field of reproduction, there is increasing interest in using mammalian stem cells to produce germ cells. Much progress has been made in in vitro germ cell differentiation [3, 44–46]. A three-dimensional induction (3d-i) system can effectively differentiate human SSCs into functional haploid sperm cells [47]. Embryonic stem cells co-cultured with *Kit* mutant testis cells, RA, and cytokines can also undergo complete meiosis and produce SLCs, and healthy offspring can be produced after ICSI [43]. However, no studies addressing the transdifferentiation of mammalian XX female stem cells into functional sperm have been reported. To our knowledge, our study is the first of its kind and also provides a strategy for dairy cattle breeding to produce only female offspring with a high-quality genetic background. The strategy involves FGSCs isolated from the ovaries of high-quality dairy cattle, without being transfected with any foreign genes, being cultured in a microenvironment composed of testis somatic cells, RA, and cytokines to obtain functional SLCs. Healthy offspring with very similar genetic backgrounds can be produced after ICSI with MII eggs derived from the ovaries of dairy cattle. This strategy will not only greatly simplify the process and shorten the time needed to breed high-quality dairy cattle, but will also increase the number of high-quality dairy cattle. However, much work needs to be done before this strategy can be implemented. First, the mechanism by which masculinizing signals induce the transdifferentiation of FGSCs remains unknown. Second, the low transdifferentiation efficiency in vitro may be due to the apoptosis or death of some FGSCs under such differentiation conditions, such as no serum, RA and other factors (Fig. 3D). The efficiency of FGSC transdifferentiation should be improved, possibly by using small-molecule compounds or other cytokines, or by modifying the culture conditions.

## Conclusion

Our findings indicate that, in mice, FGSCs without a Y chromosome can complete male meiosis and develop into functional sperm and SLCs within the testicular microenvironment and cytokine conditions. This study indicates that sperm can be produced from FGSCs without a Y chromosome, and provides a strategy for dairy cattle breeding to produce only female offspring with a high-quality genetic background.

## Methods

### Animals

We purchased *Kit*^*w/wv*^ mutant mice from the Jackson Laboratory (Bar Harbor, ME). We purchased C57BL/6 mice from SLRC Laboratory (Shanghai, China). All animal experimental procedures were approved by the institutional animal care and use committee of Shanghai Jiao Tong University (2016084), and the National Research Council Guide for Care and Use of Laboratory Animals.

### FGSC isolation and culture

We isolated and purified FGSCs from neonatal mouse ovaries (6 days old, C57BL/6) following a previously described protocol [31]. To trace cells during in vivo and in vitro transdifferentiation, FGSCs were derived from actin-GFP mice. For immunofluorescence and infection with lentivirus carrying STRA8-EGFP or PRM1-EBFP plasmids, FGSCs were derived from wild-type mice. We cultured FGSCs on SIM-6-thiogunanie-oualiain(STO) feeder cells in MEMα basal medium (Invitrogen) supplemented with 10% fetal bovine serum (FBS) (Life Technologies), 1 mM sodium pyruvate (Amresco), 2 mML-glutamine (Amresco), 50 μM β-mercaptoethanol (Biotech), 1 mMnonessential amino acids (NEAA) (Invitrogen), 20 ng/ml mouse epidermal growth factor (EGF) (PeproTech), 10 ng/ml human basic fibroblast growth factor (bFGF) (PeproTech), 10 ng/ml mouse glial cell line-derived neurotrophic factor (GDNF) (PeproTech), and 10 ng/ml ESGRO (mouse leukemia inhibitory factor, LIF) (Santa Cruz Biotechnology). FGSCs were identified by immunofluorescence staining (Positive expression of FRAGILIS, MVH, and OCT4; negative expression of PLZF.) and RT-PCR (Additional file 1: Figure S1).

### SSC isolation and culture

We isolated and purified SSCs from neonatal mouse testes (6 days old, C57BL/6) following a previously described protocol [48]. We cultured SSCs on STO feeder cells in MEMα basal medium (Invitrogen) supplemented with 10% FBS (Life Technologies), 1 mM sodium pyruvate (Amresco), 2 mM L-glutamine (Amresco), 50 μM β-mercaptoethanol (Biotech), 1 mM NEAA (Invitrogen), 20 ng/ml mouse EGF (PeproTech), 10 ng/ml human bFGF (PeproTech), 10 ng/ml mouse GDNF (PeproTech), 10 ng/ml ESGRO (Santa Cruz Biotechnology), and 100 μg/ml transferrin (Sigma). Spermatogonial stem cells were identified by immunofluorescence staining and RT-PCR (Additional file 1: Figure S2).

### Transplantation of FGSCs into recipient testes

FGSCs were derived from C57BL/6 mouse ovaries and cultured for eight passages. Then, they were digested into single-cell suspensions using 0.05% trypsin (Gibco) and suspended in phosphate-buffered saline (PBS). The FGSCs were transplanted into histocompatible *Kit*^*w/wv*^ mutant mice. Only 6- to 8-week-old male mice were used as recipients. Then, 10 µl of cell suspension containing approximately 5 × 10^7^ cells was injected into the testis reticulum of each testis.

### Histological analysis

For whole-mount observation, two to three fragments of seminiferous tubules were dissected out from a testis organ culture under a stereomicroscope, washed with PBS containing 3% (vol/vol) FBS, mounted on a glass slide, and then observed under a fluorescence microscope. For whole-mount immunofluorescence, fragments were fixed with PBS containing 4% paraformaldehyde (PFA) for 2 h at 4 °C, washed with PBS three times, blocked by PBS supplemented with 1% BSA for 1 h at room temperature, and then incubated overnight with primary antibodies in PBS at 4 °C. The next day, the tubules were washed with 0.1% Tween 20 in PBS (PBST) and incubated with secondary antibodies in PBS at room temperature for 2 h. After washing with PBST for 15 min, several pieces of seminiferous tubule were mounted on a glass slide, and then observed under a fluorescence microscope. The primary antibodies used were PLZF (1:150; Santa Cruz) and GFP (1:200; Santa Cruz).

Twenty to fifty days after transplantation, the recipient testes were removed from the mice, fixed by 4% PFA for 48 h at 4 °C, and then processed for paraffin sectioning. Sections were stained with hematoxylin and eosin (H&E). For immunofluorescence, the sections were boiled for 15 min in sodium citrate buffer for antigen retrieval. After blocking with goat serum for 30 min, each section was incubated with a primary antibody overnight at 4 °C and then washed in PBS three times. Germ cells were detected by immunofluorescence staining using an anti-MVH antibody (Abcam). Meiosis was detected by immunofluorescence using an anti-SCP3 antibody (Abcam). Post-meiosis spermatids were detected by immunofluorescence staining using an anti-ACR antibody (Santa Cruz). After washing in PBS three times, the sections were incubated with TRITC-conjugated secondary antibody (Proteintec) at a dilution of 1:200 for 1 h at 37 °C. Next, nuclei were stained with 4,6-diamidino-2-phenylindole (DAPI). Images were collected immediately using a fluorescence microscope (Leica, Germany).

### Fusion gene constructs and lentivirus infection

STRA8-EGFP was constructed by combining a 1.4 kb *Stra8* promoter with an EGFP sequence by overlap PCR [24]. The *Stra8* promoter was amplified by genomic PCR with the primers 5′-CTTGCCTCCAAGGGGGTAAGG-3′ and 5′-CGACTGCCCGTCGCAGAATA-3′. PRM1-EBFP was constructed by combining a + 652/ + 2 fragment (*Prm1* promoter, + 1 indicates the translation start site) with an EBFP sequence by overlap PCR. The + 652/ + 2 fragment was amplified by genomic PCR with the primers 5′-GTCTAGTAATGTCCAACACCT-3′ and 5′-GGTGCTGGCTTGGCCGGGAGC-3′. Both FGSCs and SSCs were infected with lentivirus carrying STRA8-EGFP or PRM1-EBFP plasmids (plvx-mcherry-N1, FENGHUISHENGWU, FH1891).

### In Vitro spermatogenesis

Testes from 2- to 7-day postpartum *Kit*^*w/wv*^ mice were harvested and extracted following a previously described protocol [49, 50]. Briefly, after euthanasia by cervical dislocation, testes were collected, decapsulated, cut into small pieces, and then dissociated with collagenase IV (1 mg/ml, Sigma) for 10 min and in 0.05% trypsin (Gibco) for 6 min at 37 °C in a shaking water bath. A single-cell suspension was obtained after filtration through a 72-µm cell strainer, and cells were collected by centrifugation. *Kit*^*w/wv*^ mouse testicular cells were directly mixed with FGSCs/SSCs at a ratio of 1:1 or FGSCs/SSCs were seeded on *Kit*^*w/wv*^ mouse testicular cells that had been cultured in Dulbecco’s modified Eagle’s medium supplemented with 10% FBS for 3 days. From day 0 to day 6, cells were cultured in MEMα (Invitrogen) containing 20% Knockout Serum Replacement (KSR) (Gibco), bone morphogenic proteins 2/4/7 (BMP-2/4/7, 20 ng/ml each; R&D Systems), retinoic acid (1 μM; Sigma), and activin A (100 ng/ml; R&D Systems). From day 7 to day 14, cells were cultured in MEMα (Invitrogen) supplemented with 20% KSR, testosterone (T, 10 mM; Acros Organics), follicle-stimulating hormone (FSH, 200 ng/ml; Sigma), and bovine pituitary extract (BPE, 50 mg/ml; Stem Cell). The medium was changed every day. Cells were cultured in 5% CO_2_ at 37 °C.

### RT-PCR analysis

Total mRNA were extracted using Trizol reagent (Qiagen) following the manufacturer’s instructions. cDNA was reverse-transcribed from total mRNA using M-MLV reverse transcriptase (Takara) and was then used for PCR analysis. Primer information is listed in Additional file 1: Table S1.

### Spermatocyte surface spreading

A previously described drying-down technique was used to spread spermatocytes generated by in vitro spermatogenesis [51]. Briefly, the cells were digested into single-cell suspensions using 0.05% trypsin (Gibco) and washed with PBS. Then, the cells were placed into freshly prepared buffer (30 mM Tris, pH 8.2; 50 mM sucrose; 17 mM citric acid; 5 mM EDTA; 0.5 mM DTT; 0.1 mM PMSF, pH 8.2–8.4) for 30–60 min. Subsequently, the cells were collected by centrifugation (350 × g, 5 min) and put into 100 mM sucrose (pH 8.2) for 20 min. Finally, the cell suspensions were placed on slides containing 1% PFA, pH 9.2, and 0.15% Triton X-100, which were then dried overnight in a closed box with high humidity.

### Immunofluorescence microscopy

For immunofluorescence, cultured cells were fixed with 4% PFA in PBS (pH 7.4) for 30 min, washed three times with PBST, followed by incubation with 1% Triton X-100 in PBS for 15 min (for cells spread with PMSF, this step was omitted). Cells or slides were blocked by PBS containing 5% goat serum for 1 h at room temperature, and then incubated with primary antibodies diluted in PBS overnight at 4 °C. Primary antibodies were diluted with PBS as follows: MVH (1:200; Abcam), FRAGILIS (1:100; Abcam), OCT4 (1:100; Abcam), PLZF (1:100; Santa Cruz), SCP3 (1:50; Abcam), SCP1 (1:50; Santa Cruz), and ACROSIN (1:50; Santa Cruz). After incubation, the slides or cells were washed with PBST and then incubated with TRITC- or FITC-conjugated secondary antibodies (1:200; Invitrogen) in PBS at 37 °C, followed by washing and staining with DAPI.

### DNA content staining for FACS analysis

Differentiated cells were digested into single-cell suspensions using 0.05% trypsin (Gibco), fixed with 70% ethanol at 4 °C for 30 min, and then passed through a cell strainer with a 40 µm pore size. Cells were resuspended in PBS supplemented with 3% FBS and then incubated in PBS containing 0.2 mg/ml RNase A, 0.02 mg/ml propidium iodide, and 0.1% Triton X-100 for 15 min at 37 °C. The cell suspension was analyzed and sorted by flow cytometry using a FACSCalibur (Becton Dickinson).

### Low-input Hi-C Library generation

We performed in situ Hi-C using a modified protocol based on previous reports [52–55]. Briefly, ~ 10,000 cells were fixed with 1% PFA for 10 min, and then fixation was stopped with glycine. Lysed cells were digested with *Dpn*II (NEB, R0543M), and chromatin ends were filled and marked with biotin-14-dATP (Life Technologies). The purified DNA was fragmented with *Alu*I (NEB, R0137V) and size-selected. The DNA fragments were enriched via the biotin pull-down assay. A DNA Library Prep Kit (Vazyme, ND607-01) was used to construct a standard DNA library, which was sequenced using the Illumina sequencing platform. For each stage, we sequenced 8.0–9.0 billion Hi-C reads, resulting in 73–386 million pairwise chromatin contacts (Additional file 1: Table S2).

### μChIP-seq

We performed μChIP-seq by using approximately 10,000 cells per reaction and two replicates for each stage. Cells were isolated by FACS based on EGFP- or EBFP-positive expression. Briefly, cells were fixed in 1% PFA, which was terminated with 125 mM glycine. After centrifugation (550 × g), the supernatant was carefully aspirated and the pellet was resuspended in ChIP Lysis Buffer (200 μl) (Beyotime) for 10 min. The lysate was then sonicated to shear DNA to an average fragment size of 200–500 bp. From each sonicated sample, 50 μl was retained as the input. Then, the rest of the sample was reacted in RIPA buffer (Beyotime) at 4 °C overnight with H3K4me3 (ab8580; Abcam) or H3K27me3 (07–449; Millipore) antibodies, which were pre-bonded to paramagnetic beads (Promega). The beads were washed by RIPA buffer and the tubes were placed in a magnetic rack. DNA was isolated and purified using proteinase K (Merck), phenol–chloroform-isoamylalcohol (25:24:1) (Bio-Rad), chloroform-isoamylalcohol (24:1) (Bio-Rad), 100% ethanol, and 75% ethanol.

In accordance with the manufacturer’s instructions, we generated sequencing libraries by using a VAHTS Universal DNA Library Prep Kit (Vazyme, ND607-01). We then sequenced the libraries using the Illumina sequencing platform.

### Bisulfite sequencing analysis

Genomic DNA was extracted from mouse tail, FGSCs, SSCs, SLCs or sperm. Genomic DNA (15–20 ng) was then processed using the EZ DNA Methylation-Gold Kit™ (ZYMO Research), in accordance with the manufacturer’s instructions. The bisulfite-treated genomic DNA (4–5 μl) was amplified by PCR, the primers for which are shown in Additional file 1: Table S1. The PCR fragments were cloned in pMD19-T, sequenced, and analyzed using QUMA software.

### Intracytoplasmic sperm injection (ICSI) and embryo transfer

The injection procedure was performed as previously described [25]. Briefly, BDF1 mice were used as oocyte donors. fSLCs (GFP-positive and haploid cells) were sorted using FACS and sperm were taken from the epididymal tail. Cytoplasm was removed from these sorted fSLCs, which were then injected together with sperm into cumulus-free oocytes. GFP was monitored under fluorescence microscopy at different development stages. Embryos that reached the two-cell stage after culture for 24 h were transferred to the oviduct of embryonic day (E)0.5 CD1 pseudo-pregnant females or further cultured in KSOM_AA_ medium until the blastocyst stage. Full-term pups were delivered naturally.

### Southern blotting

Genomic DNA of mouse tails was extracted. After digestion by *EcoR*I, samples were loaded onto a 1% agarose gel and run at 35 V for 17 h. After denaturation and neutralization, DNA was transferred by capillary action to a nylon membrane (Amersham Pharmacia Biotech, Arlington Heights, IL, USA). After prehybridization, hybridization was carried out with a GFP probe. Signals were then detected using a CDP-Start kit (GE Healthcare).

### Karyotype analysis

Cultured cells were treated with colchicine (100 ng/mL, Sangon) for 2.5 h and hypotonically treated with 75 mM KCl for 40 min at 37 °C. Then, the cells were fixed in methanol-acetic acid (3:1) overnight at 4 °C. The fixed cells were then dropped onto a glass slide from a height of approximately 1 m, air-dried, and stained with Giemsa (Sangon).

### Statistical analysis

To evaluate the significance of differences between groups, one-way ANOVA or *t*-test was used. *P* < 0.05 was considered statistically significant.

### Hi-C data mapping

Hi-C data were processed using Hi-CPro (version 2.11.1), as previously described [56]. Briefly, paired-end reads were aligned to the mouse reference genome (mm9) using Bowtie2 (version 2.3.5.1). Then, we discarded unmapped reads, singletons, self-circle ligations, and PCR duplicates, and obtained the valid reads with which to build the matrix for downstream analysis.

### A/B compartment identification

A/B compartment analysis was performed at 400 kb resolution using R package (HiTC) [57] with the pca. Hi-C function in R on the matrix. A positive value represented the A compartment, while negative value represented the B compartment.

### TAD calling

The boundaries were calculated using the directional index (DI) value at 20 kb resolution, as previously described [58]. TAD boundaries were defined as those < 400 kb.

### ChIP-seq analysis

The paired-end reads of ChIP-seq were mapped to the mouse reference genome (mm9) using Bowtie2. Duplicate reads were removed with SAMtools (version 1.9). Then, the reads were allocated into 50-bp bins and normalized to 1 × genome coverage. MACS2 was used to call peaks with the adjusted *P*-value cut-off set as 0.01.

## Supplementary Information

**Additional file 1:** **Table S1.** Oligonucleotide primer sequences. **Table S2.** Valid Hi-C data. **Figure S1.** Characterization and identification of FGSCs. **Figure S2.** Characterization and identification of SSCs. **Figure S3.** FGSCs injected into *Kit*^*w/wv*^ mouse seminiferous tubules transdifferentiate into male germ cells and express PLZF in a testicular microenvironment. **Figure S4.** PLZF was expressed in GFP positive cells by Immunofluorescence analysis. **Figure S5.** In vitro induction of meiosis in SSCs and formation of SLCs derived from SSCs. **Figure S6.** Comparison of in vitro transdifferentiation of FGSCs with in vitro differentiation of SSCs. **Figure S7.** Comparison between Hi-C seq and ChIP seq biological replicates. **Figure S8.** k-means clustering of H3K4me3 and H3K27me3 at all TSSs in different cell states and enriched GO terms. **Figure S9.** The number of A/B compartments, genes in TADs, and genes outside of TADs in different cell states based on four clusters. **Figure S10.** Methylation and expression analysis of imprinted genes.

**Additional file 2.** Graphic abstract.

## Data Availability

All data needed to evaluate the conclusions in the paper are present in the paper and/or the Additional files 1. Raw sequence data of Hi-C and ChIP-seq have been submitted to the NCBI Sequence Read Archive under Accession number GSE163931. Additional data related to this paper may be requested from the authors.
