# Supplementary material for: Offspring production of haploid spermatid-like cells derived from mouse female germline stem cells with chromatin condensation
Source: Cell Biosci. 2022 Jan 4;12:5. doi: 10.1186/s13578-021-00697-z (PMC8729121; doi:10.1186/s13578-021-00697-z)
Supplement: Supplementary file 1 — Additional file 1: Table S1. Oligonucleotide primer sequences. Table S2. Valid Hi-C data. Figure S1. Characterization and identification of FGSCs. Figure S2. Characterization and identification of SSCs. Figure S3. FGSCs injected into Kitw/wv mouse seminiferous tubules transdifferentiate into male germ cells and express PLZF in a testicular microenvironment. Figure S4. PLZF was expressed in GFP positive cells by Immunofluorescence analysis. Figure S5. In vitro induction of meiosis in SSCs and formation of SLCs derived from SSCs. Figure S6. Comparison of in vitro transdifferentiation of FGSCs with in vitro differentiation of SSCs. Figure S7. Comparison between Hi-C seq and ChIP seq biological replicates. Figure S8. k-means clustering of H3K4me3 and H3K27me3 at all TSSs in different cell states and enriched GO terms. Figure S9. The number of A/B compartments, genes in TADs, and genes outside of TADs in different cell states based on four clusters. Figure S10. Methylation and expression analysis of imprinted genes. [file 13578_2021_697_MOESM1_ESM.pdf]

Supporting information

Offspring Production of Haploid Spermatid-Like Cells Derived from Mouse Female Germline Stem Cells with Chromatin Condensation

Xiaopeng Hu#, Hu Wang#, Geng.G Tian#, Changliang Hou#, Bo Xu, Xinyan Zhao, Yongqiang Zhao, Qian Fang, Xinyue Li, Lin He, Xuejin Chen, Shangang Li and Ji Wu**

Supplemental Tables

Table S1 Oligonucleotide primer sequences

Gene	Forward	Reverse
	**For RT-PCR and qRT-PCR**	
Oct4	CCCGGAAGAGAAAGCGAACT	GGAAAGGTGTCCCTGTAGCC
Fragilis	GTTATCACCATTGTTAGTGTCATC	AATGAGTGTTACACCTGCGTG
Mvh	GTGGAAATACTGGCAGAGCG	CTGAAGCTGGGAGGCACATA
Stella	CCCAATGAAGGACCCTGAAAC	AATGGCTCACTGTCCCGTTCA
Dazl	GTGTGTCGAAGGGCTATGGAT	ACAGGCAGCTGATATCCAGTG
Blimp1	CGGAAAGCAACCCAAAGCAATAC	CCTCGGAACCATAGGAAACATTC
Plzf	TTCAGCCTCAAGCACCAGTT	GGGCAGTATTCCGTGCAGAT
Gfra1	GGAGGCCTTGAAGCAGAAGT	AACGGGACTGCCCGGAATA
Etv5	CTGGGGAACGCTACGTCTAC	CCAGGAGGTAAGCAGGGTTG
Stra8	TCACAGCCTCAAAGTGGCAGG	GCAACAGAGTGGAGGAGGAGT
Scp1	GACAACGGCCCAGGAGGCA	TCTGCGGTTTCACGGCGGA
Scp2	GACACTGAAACCGAATGTGGA	TGTGGGTCTTGGTTGTCCTTT
Scp3	GAGCCGCTGAGCAAACATCTA	ATATCCAGTTCCCACTGCTGC
Tnp1	AGCCGCAAGCTAAAGACTCA	CTCTCTTGACGCCCTTGTGA
Haprin	TATGCGTTCCGAGTGAGAGC	AGGTGCTCAGTGTTGTAGCC
Prm1	AGCAAAAGCAGGAGCAGATG	CTTGCTATTCTGTGCATCTAG
Acrosin	CGGAGTCTACACAGCCACCT	GCATGAGTGATGAGGAGGTT
H19	CATGTCTGGGCCTTTGAA	TTGGCTCCAGGATGATGT
Gtl2	TTGCACATTTCCTGTGGGAC	AAGCACCATGAGCCACTAGG
Dlk1	CTGGCGGTCAATATCATCTTCC	GAGGAAGGGGTTCTTAGATAGCG
Grb10	TCCAAGTGGAGAGTACCATGC	TACGGATCTGCTCATCTTCG
Snrpn	TGCTACGTGGGGAGAACTTG	CCTGGGGAATAGGTACACCTG
Plagl1	CAAAGCCTTCGTCTCCAAGTAT	GTCCTTCCGGTTGAATGTCTT
Gapdh	AGGTCGGTGTGAACGGATTTG	TGTAGACCATGTAGTTGAGGTCA
	**PCR Probe For Southern blot**	
GFP-probe	AACTCCAGCAGGACCATGTG	ATGGCCGACAAGCAGAAGAA
	**For Bisulfite sequencing PCR**	
Peg10	GTAAAGTGATTGGTTTTGTATTTTTAAGTG	TTAATTACTCTCCTACAACTTTCCAAATT
H19-inside	GTAAGGAGATTATGTTTATTTTTGG	CCTCATTAATCCCATAACTAT
H19-outside	GAGTATTTAGGAGGTATAAGAATT	ATCAAAAACTAACATAAACCCCT
Igf2r	TTAGTGGGGTATTTTTATTTGTATGG	AAATATCCTAAAAATACAAACTACACAA

Table S2 Valid Hi-C data

Cell Type	RE	Replicate	Total Reads	Unique Read pairs (%)	Valid HiC pairs (%)	Duplication Remaining Read pairs	Cis short-range contacts(<20kb)(%)	Cis long-range contacts(<20kb)(%)	Trans Contacts (%)
	Mbol	R1	443540819	178505556	141608179	18252629	2362598	9220111	6669920
STRA8-tGCs	Mbol	R2	453139713	163846061	131657165	18493985	2378653	9297939	6817393
	Mbol	Comb	896680532	342351617	273265344	36746614	4741251	18518050	13487313
	Mbol	R1	436686752	212012657	189633058	158929277	23141608	97504360	38283309
STRA8-mGCs	Mbol	R2	447941340	219568623	195560080	160359931	23250043	98037945	39071943
	Mbol	Comb	884628092	431581280	385193138	319289208	46391651	195542305	77355252
	Mbol	R1	364809945	204040518	27915308	24353955	819710	5665056	17869189
PRM1-tGCs	Mbol	R2	441867824	262754408	45779840	41652392	1371344	10119479	30161569
	Mbol	Comb	806677769	466794926	73695148	66006347	2191054	15784535	48030758
	Mbol	R1	377764719	27443835	102509458	91728670	2270136	27349942	62108592
PRM1-mGCs	Mbol	R2	459683367	261643709	170975209	155170297	3953603	47929621	103287073
	Mbol	Comb	837448086	289087544	273484667	246898967	6223739	75279563	165395665

Supplemental Figures

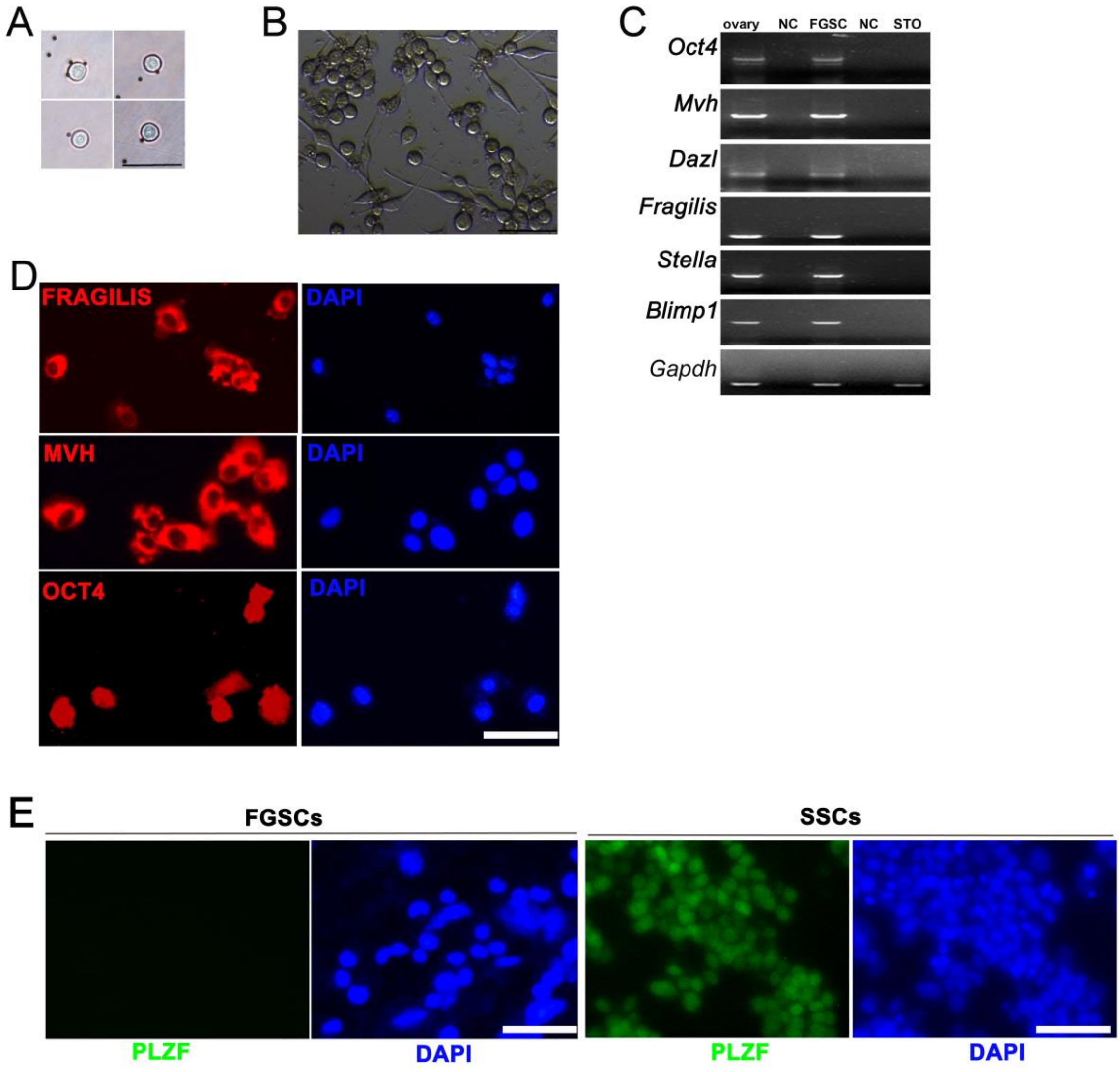

Figure S1 Characterization and identification of FGSCs.

(A) Morphology of neonatal mouse FGSCs isolated by magnetic beads pre-bound with MVH antibody.

(B) Morphology of neonatal mouse FGSCs after eight passages.

(C) FGSCs were identified by RT-PCR. cDNA from ovarian tissues was used as a positive control. STO cells were used as a negative control. NC: H_2O.

(D) FGSCs were identified by FRAGILIS, MVH and OCT4 immunofluorescence. Scale bar, 50 μm.

(E) FGSCs and SSCs were immunofluorescence staining with PLZF antibody. Scale bar, 50 μm.

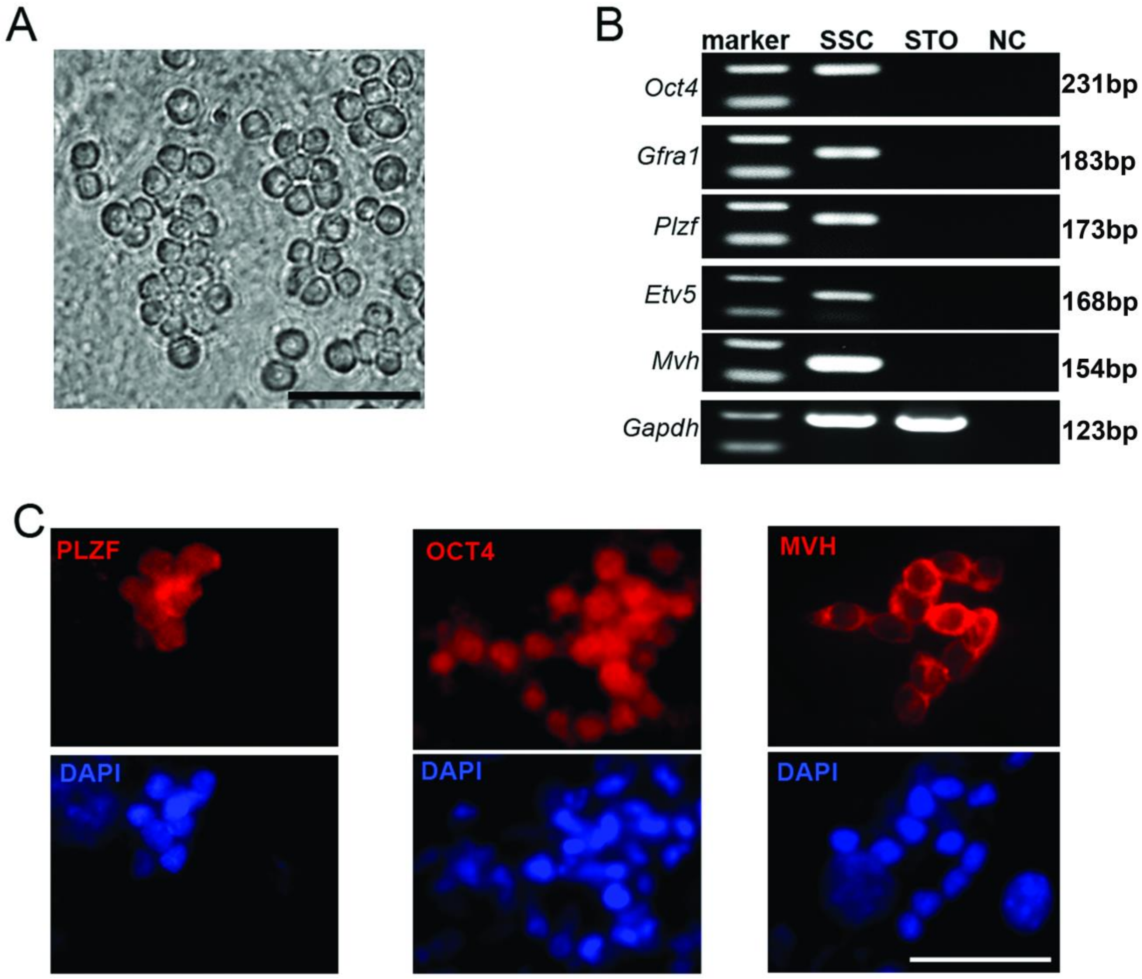

Figure S2 Characterization and identification of SSCs.

(A) Morphology of neonatal mouse SSCs after long-term culture.

(B) SSCs were identified by RT-PCR. STO cells were used as a negative control. NC: H_2O.

(C) SSCs were identified by PLZF, MVH and OCT4 immunofluorescence. Scale bar, 50 μm.

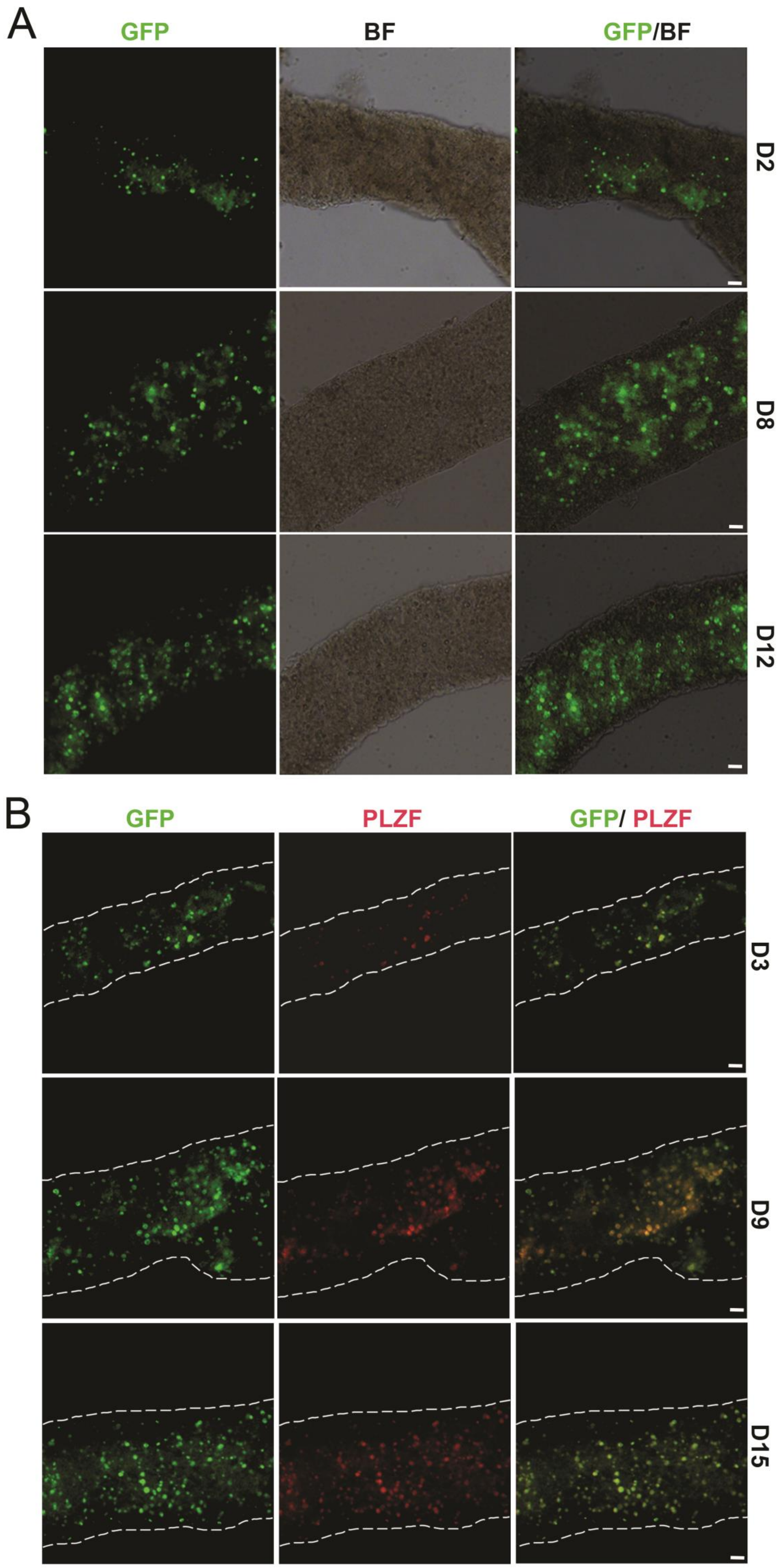
A
GFP
BF
GFP/BF
D2
D8
D12
B
GFP
PLZF
GFP/ PLZF
D3
D9
D15

Figure S3 FGSCs injected into *Kit*$^{W/WV}$ mouse seminiferous tubules transdifferentiate into male germ cells and express PLZF in a testicular microenvironment.

(A) Immunofluorescence analysis of GFP-positive cells in a recipient seminiferous tubule after culturing the recipient testis fragment *in vitro* for different times. **(B)** Dual immunofluorescence analysis of GFP-PLZF double-positive cells after culturing the recipient testis fragment *in vitro* for different times. Scale bar, 50 μm.

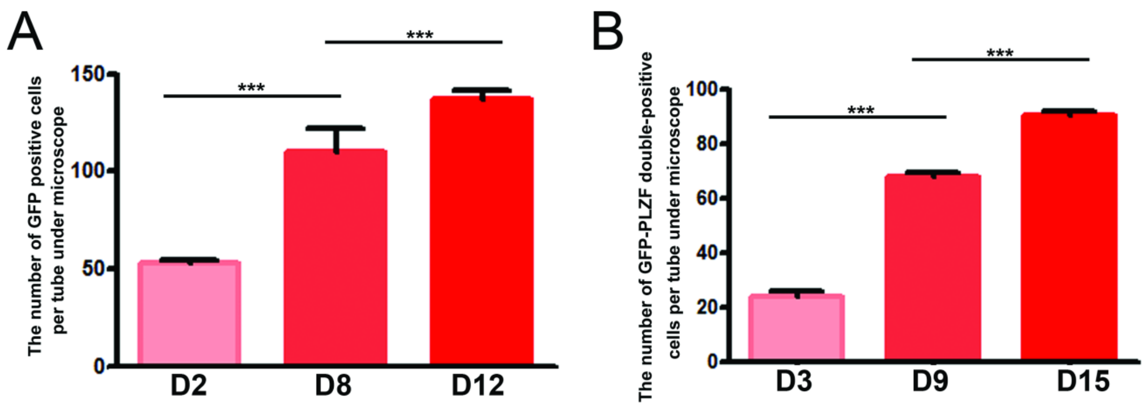

Figure S4 PLZF was expressed in GFP positive cells by Immunofluorescence analysis. **(A)** The number of GFP positive cells per tube under microscope. **(B)** The number of GFP-PLZF double-positive cells per tube under microscope.****P*<0.001.

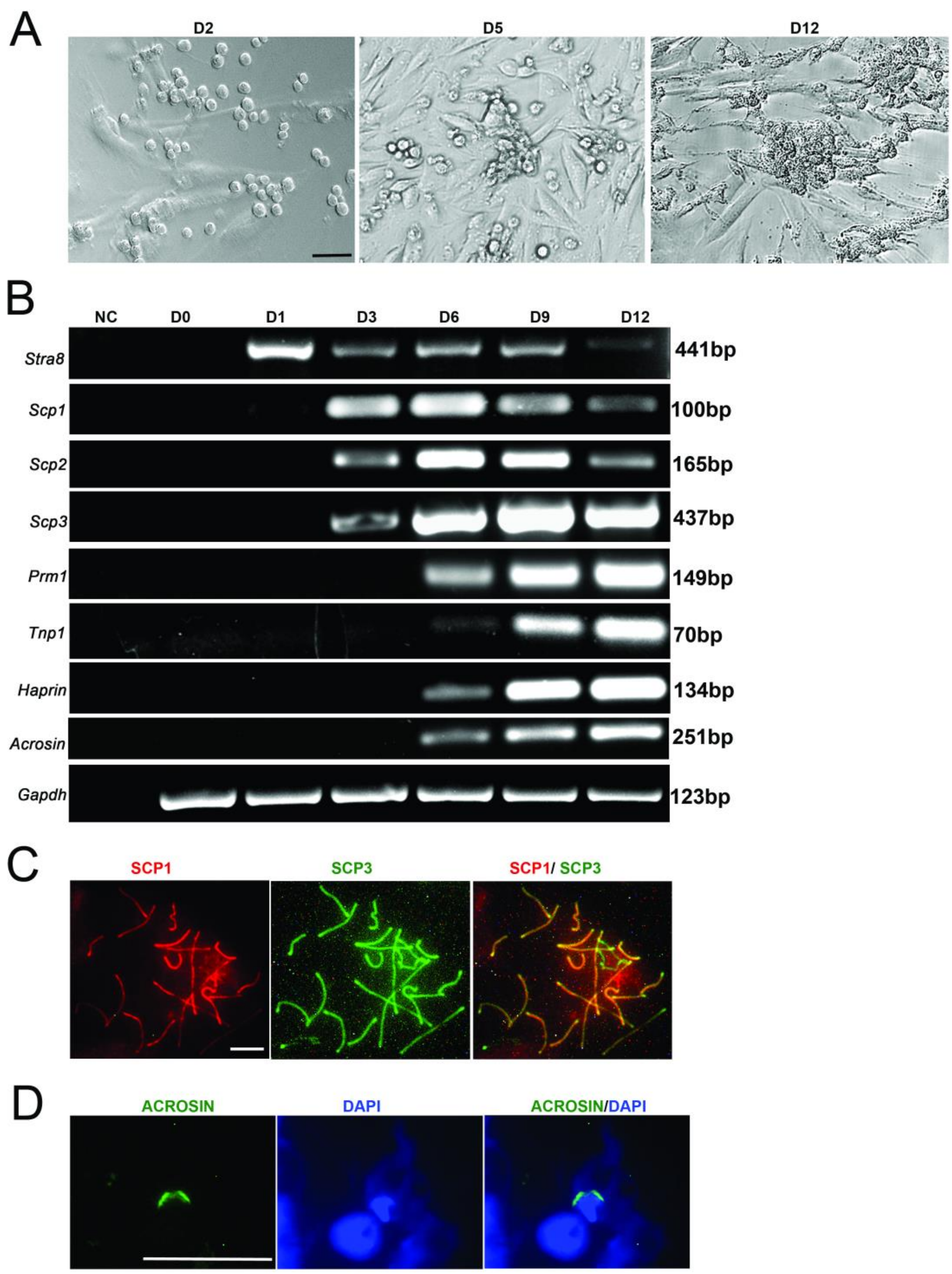

Figure S5 ***In vitro*** **induction of meiosis in SSCs and formation of SLCs derived from SSCs.**

(A)SSCs induced by *Kit*$^{w/wv}$ testicular cells, RA and cytokines were observed at different time points. **(B)** Transcripts of meiotic factors and haploid spermatid markers were detected by RT-PCR at different time points. **(C)** Chromosomal synapsis of meiosis was assessed by SCP1 and SCP3 immunofluorescence. **(D)** SLCs were confirmed by ACR immunostaining. NC: negative control. Scale bar, 50 μm.

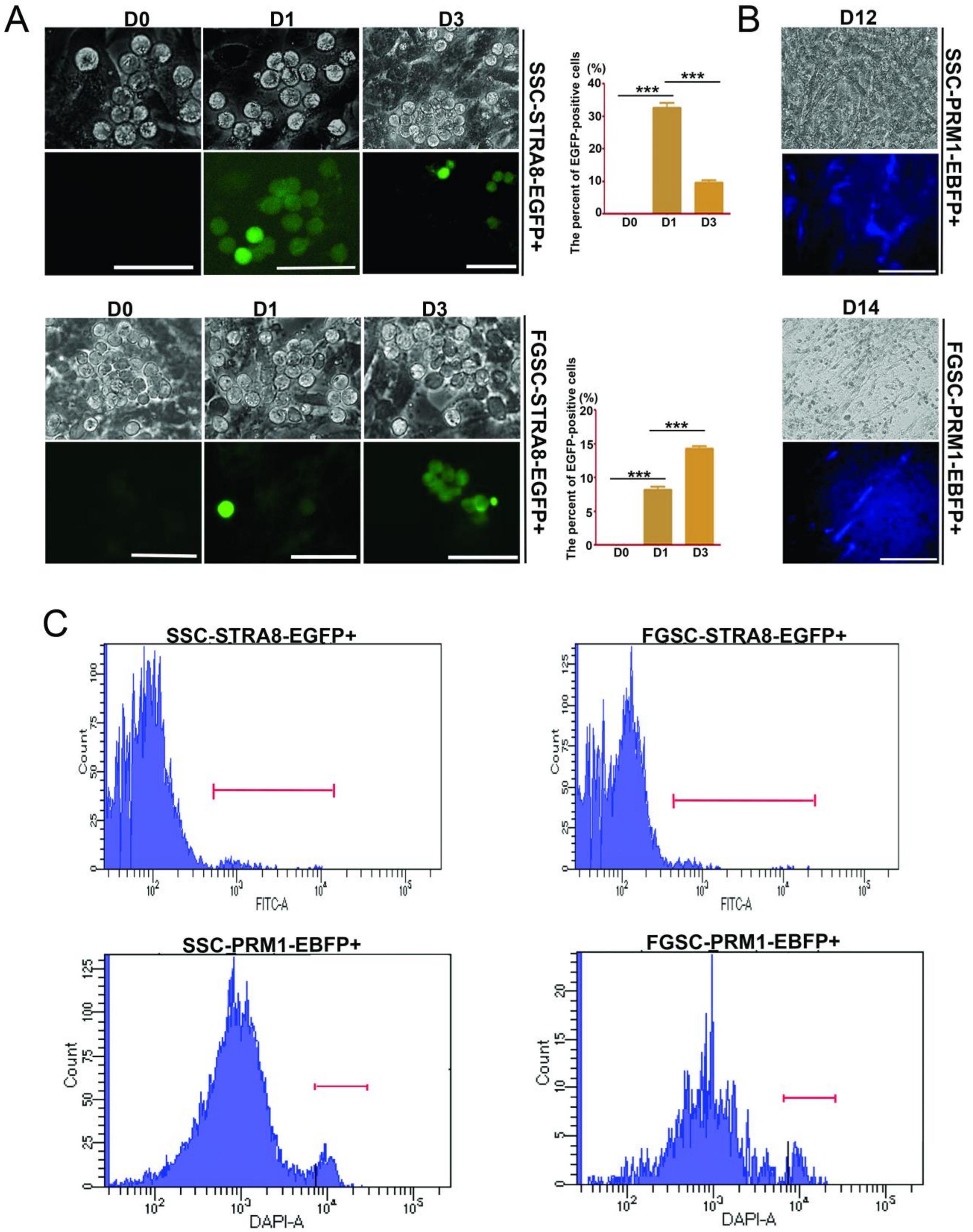

Figure S6 Comparison of *in vitro* transdifferentiation of FGSCs with *in vitro* differentiation of SSCs.

(A) Comparison of *in vitro* transdifferentiation of FGSCs with *in vitro* differentiation of SSCs with STRA8-EGFP plasmid. **(B)** Comparison of *in vitro* transdifferentiation of FGSCs with *in vitro* differentiation of SSCs with PRM1-EBFP plasmid. **(C)** STRA8-EGFP- and PRM1-EBFP-positive cells were sorted by FACS. Scale bar, 50 μm. ***P<0.001.

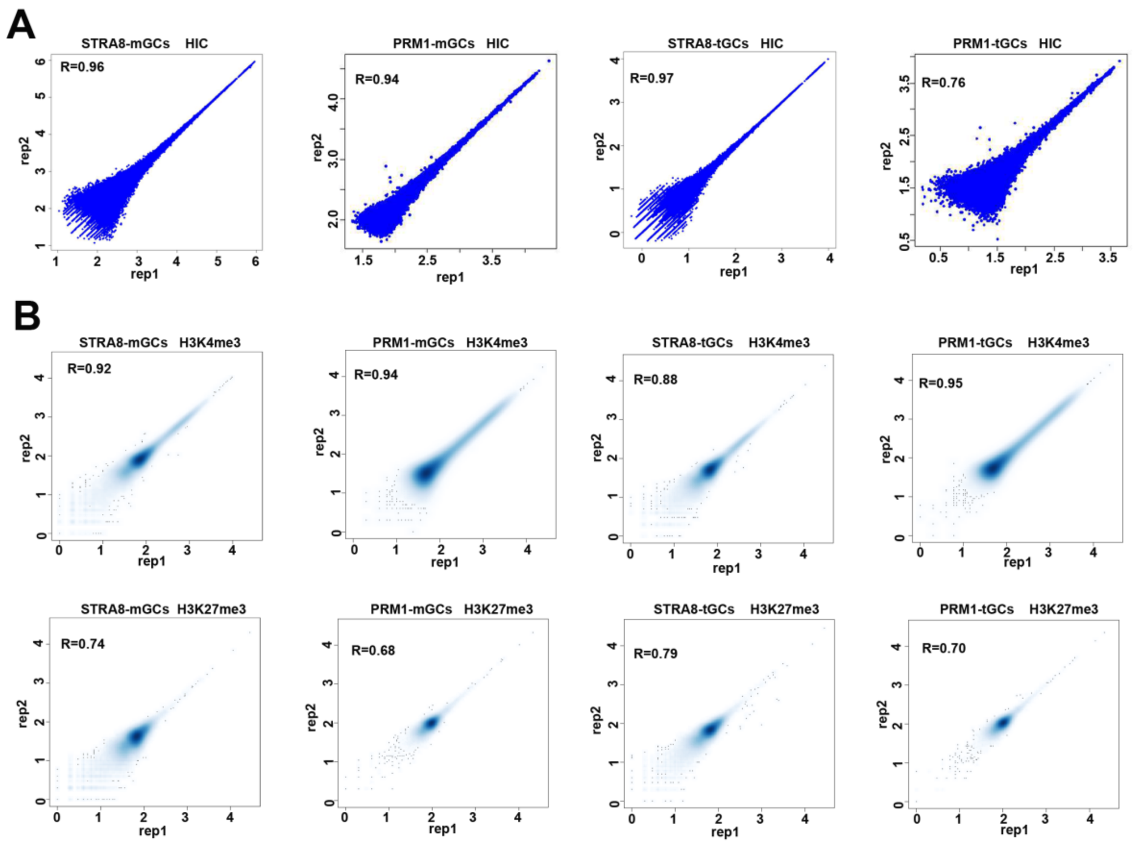

Figure S7 Comparison between Hi-C seq and ChIP seq biological replicates.

(A) Comparison between biological replicates resulted in highly reproducible Hi-C maps.

(B) Comparison between biological replicates resulted in highly reproducible ChIP-seq data.

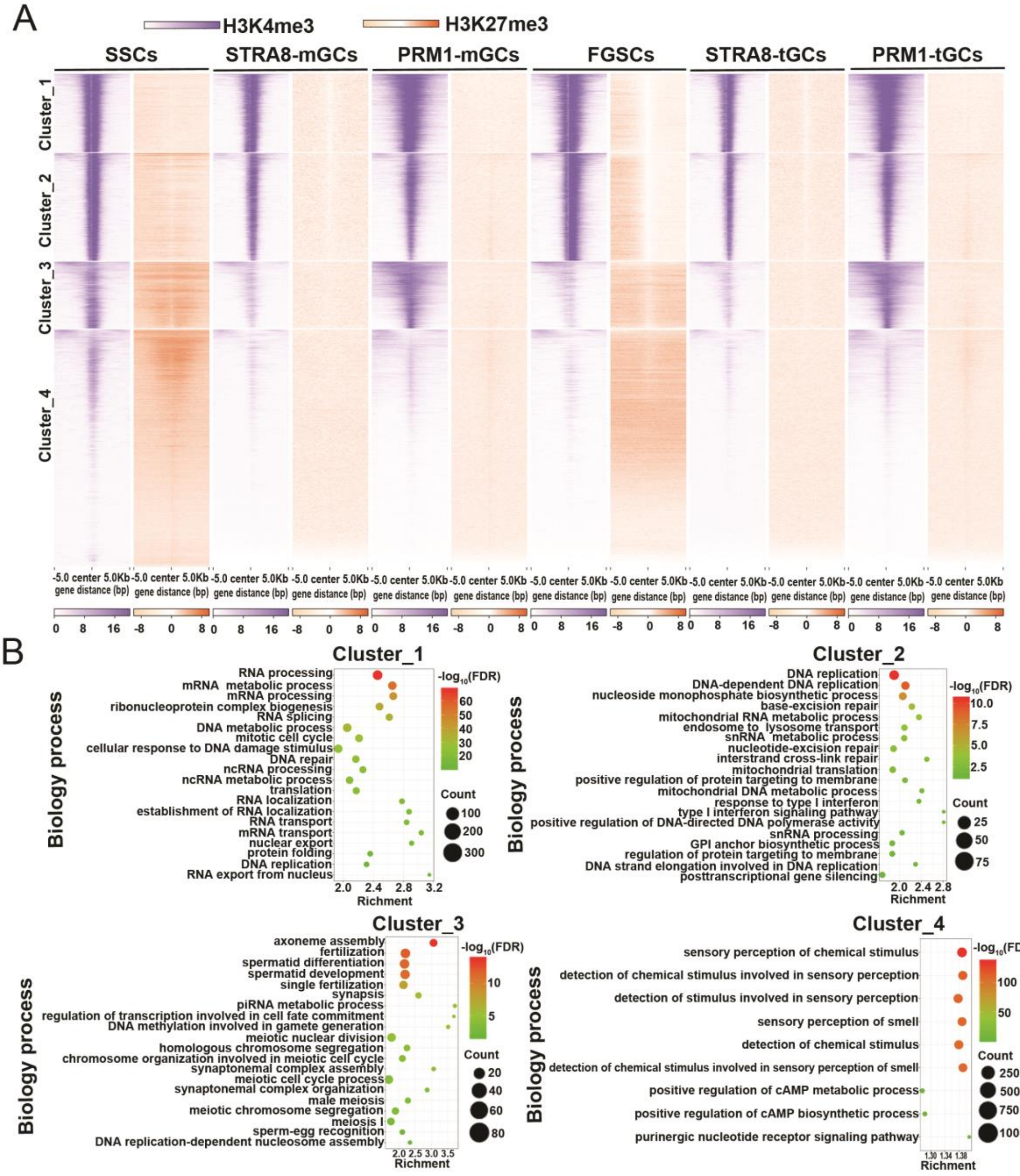

Figure S8 ***k-means*** **clustering of H3K4me3 and H3K27me3 at all TSSs in different cell states and enriched GO terms.**

(A) *k-means* clustering in heatmaps of H3K4me3 and H3K27me3 ChIP-Seq signals in different cell states (-5 kb to +5 kb). **(B)** Representative enriched GO terms of different cluster genes.

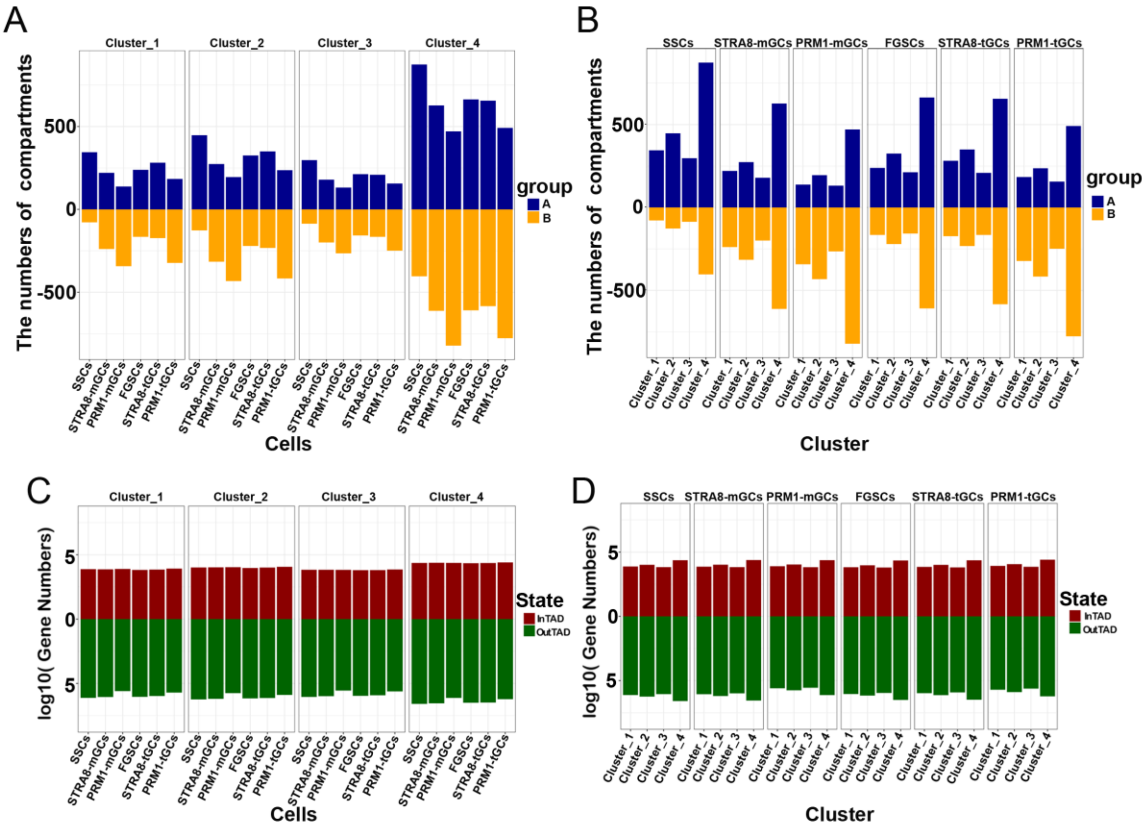

Figure S9 The number of A/B compartments, genes in TADs, and genes outside of TADs in different cell states based on four clusters.

(A) The number of A/B compartments in different cell states based on four clusters. (Blue represents A compartment; yellow represents B compartment).

(B) The number of A/B compartments of four clusters in different cell states. (Blue represents A compartment; yellow represents B compartment).

(C) The number of genes in different cell states located in TADs or outside of TADs in four clusters. (red represents within a TAD; green represents outside of a TAD).

(D) The number of genes of four clusters located in TADs or outside of TADs in different cell states. (red represents within a TAD; green represents outside of a TAD).

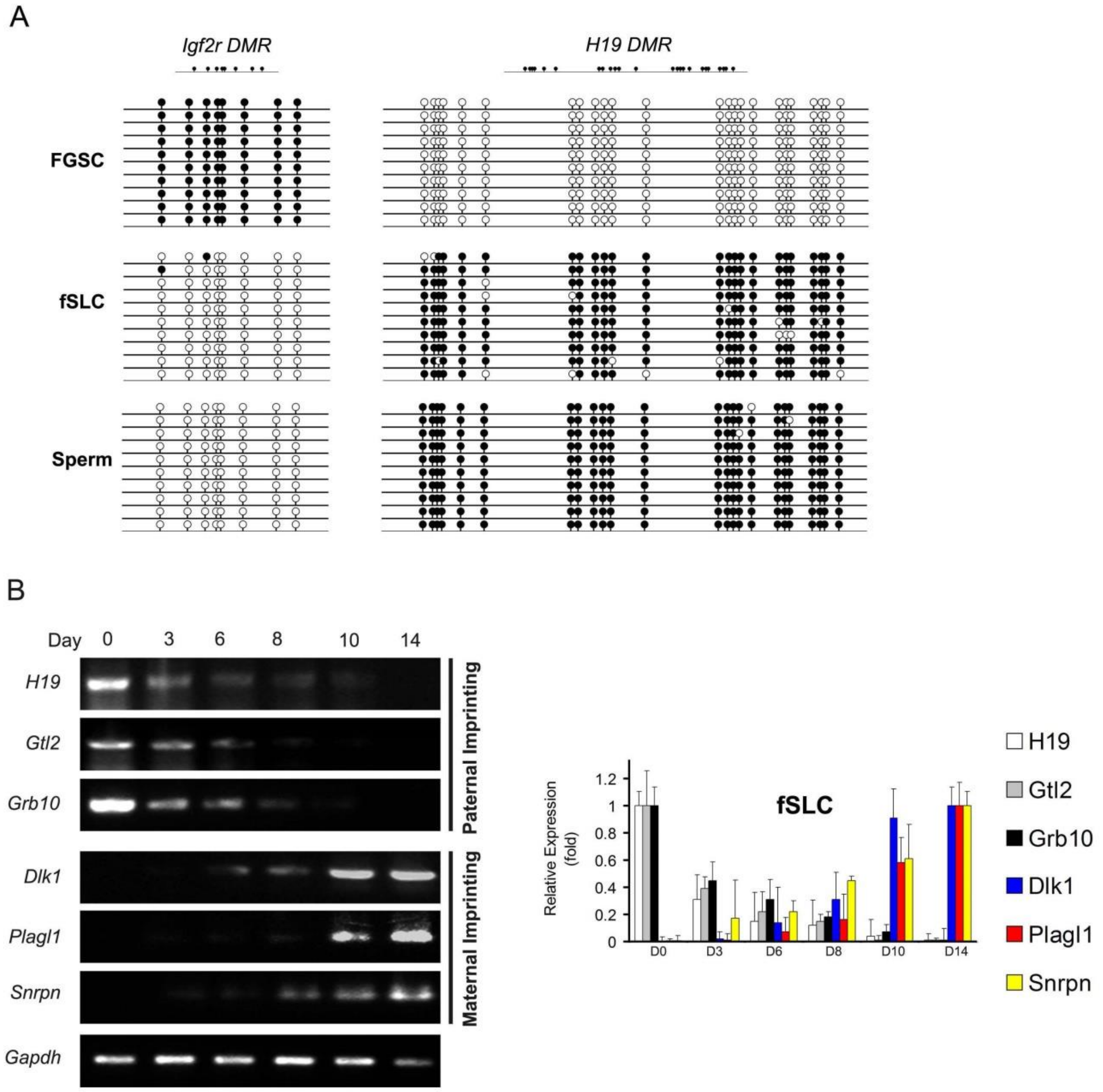

Figure S10 Methylation and expression analysis of imprinted genes.

(A) Methylation status of DMRs in fSLCs. A maternally imprinted region (*Igf2r*) and a paternally imprinted region (*H19*) were examined by bisulfite genomic sequencing. White circles indicate unmethylated CpGs and black circles indicate methylated CpGs. **(B)** Expression of imprinted genes in fSLCs as detected by RT-PCR, including paternally imprinted genes (*H19*, *Gtl2* and *Grb10*) and maternally imprinted genes (*Dlk1*, *Plagl1* and *Snrpn*). **(C)** Relative expression of imprinted genes during in vitro trandifferentiation of FGSCs (fSLCs) in different differentiation periods (mean ± SEM, n=3).
