# Supplementary figures and images for: Offspring production of haploid spermatid-like cells derived from mouse female germline stem cells with chromatin condensation
Source: Cell Biosci. 2022 Jan 4;12:5. doi: 10.1186/s13578-021-00697-z (PMC8729121; doi:10.1186/s13578-021-00697-z)

*in vivo*

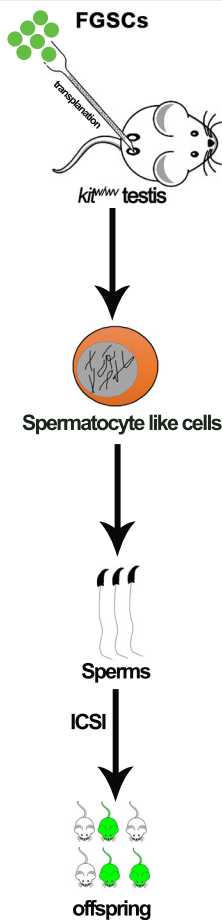

*in vitro*

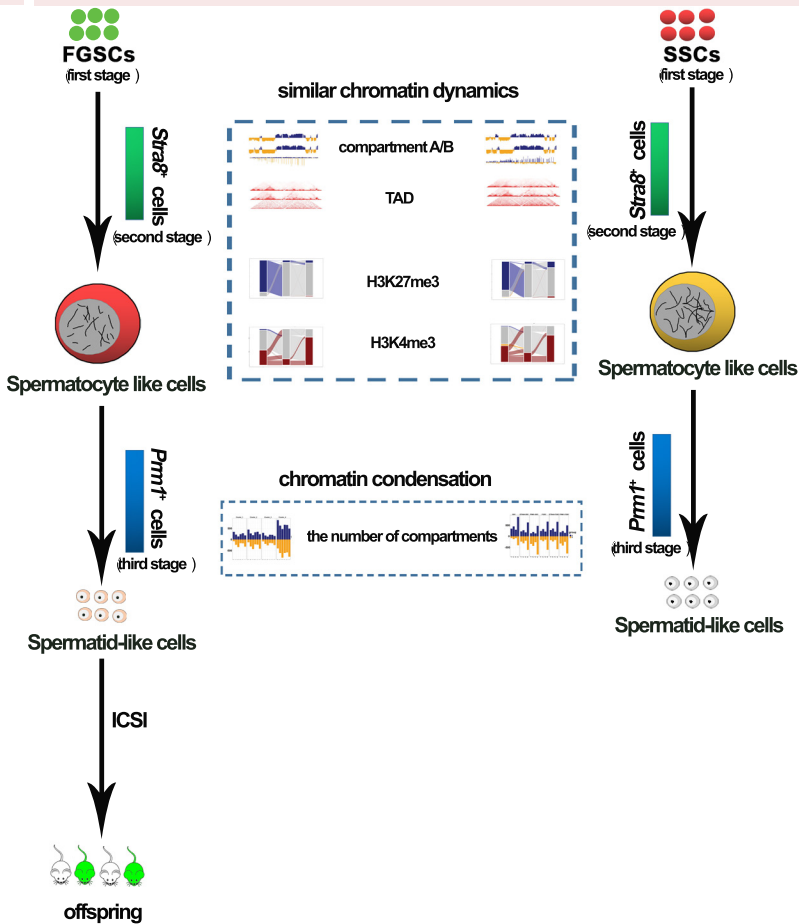

Supplement: Supplementary file 2 — Additional file 2. Graphic abstract. [file 13578_2021_697_MOESM2_ESM.pdf]
